# Favorable efficacy and reduced acute neurotoxicity by antisense oligonucleotides with 2′,4′ -BNA/LNA with 9-(aminoethoxy)phenoxazine

**DOI:** 10.1016/j.omtn.2024.102182

**Published:** 2024-04-02

**Authors:** Taiki Matsubayashi, Kotaro Yoshioka, Su Su Lei Mon, Maho Katsuyama, Chunyan Jia, Takao Yamaguchi, Rintaro Iwata Hara, Tetsuya Nagata, Osamu Nakagawa, Satoshi Obika, Takanori Yokota

## Main text

(Molecular Therapy: Nucleic Acids *32*, 1–14; March 2024)

In the originally published version of this article, part of the authorship information for Taiki Matsubayashi was incorrect. This error occurred due to an omission from the manuscript during proofreading. The equal contribution footnote for Kotaro Yoshioka and Su Su Lei Mon should have also been applied for Taiki Matsubayashi, meaning that these three authors contributed equally.

This error has been corrected in the article online.

