# Supplementary material for: Favorable efficacy and reduced acute neurotoxicity by antisense oligonucleotides with 2′,4′-BNA/LNA with 9-(aminoethoxy)phenoxazine
Source: Mol Ther Nucleic Acids. 2024 Mar 18;35(2):102161. doi: 10.1016/j.omtn.2024.102161 (PMC11229412; doi:10.1016/j.omtn.2024.102161)
Supplement: Document S1. Figures S1–S3 and Table S1 [file mmc1.pdf]

OMTN, Volume *35*

Supplemental information

Favorable efficacy and reduced acute neurotoxicity by antisense oligonucleotides with 2′,4′-BNA/LNA with 9-(aminoethoxy)phenoxazine

Taiki Matsubayashi, Kotaro Yoshioka, Su Su Lei Mon, Maho Katsuyama, Chunyan Jia, Takao Yamaguchi, Rintaro Iwata Hara, Tetsuya Nagata, Osamu Nakagawa, Satoshi Obika, and Takanori Yokota

Table S1. Abbreviation in this study.

Abbreviation	Spell out
**Gene name**	
Actb	actin beta
Malat1	metastasis associated in lung adenocarcinoma transcript-1
Mapt	microtubule-associated protein tau
**Chemical modification**	
2',4'-BNA/LNA	2'-O,4'-C-methylene-bridged nucleic acid/locked nucleic acid
BNAP-AEO	2',4'-BNA/LNA with a 9-(aminoethoxy)phenoxazine
G-clamp	9-(aminoethoxy)phenoxazine
MOE	2'O-methoxyethyl RNA
PO linkage	phosphodiester linkage
PS linkage	phosphorothioate linkage
**Others**	
ASO	antisense oligonucleotide
CNS	central nervous system
CSF	cerebrospinal fluid
DMEM	Dulbecco’s modified minimal essential medium
FBS	fetal bovine serum
FDA	US Food and Drug Administration
ICV	intracerebroventricular
T_m	melting temperature

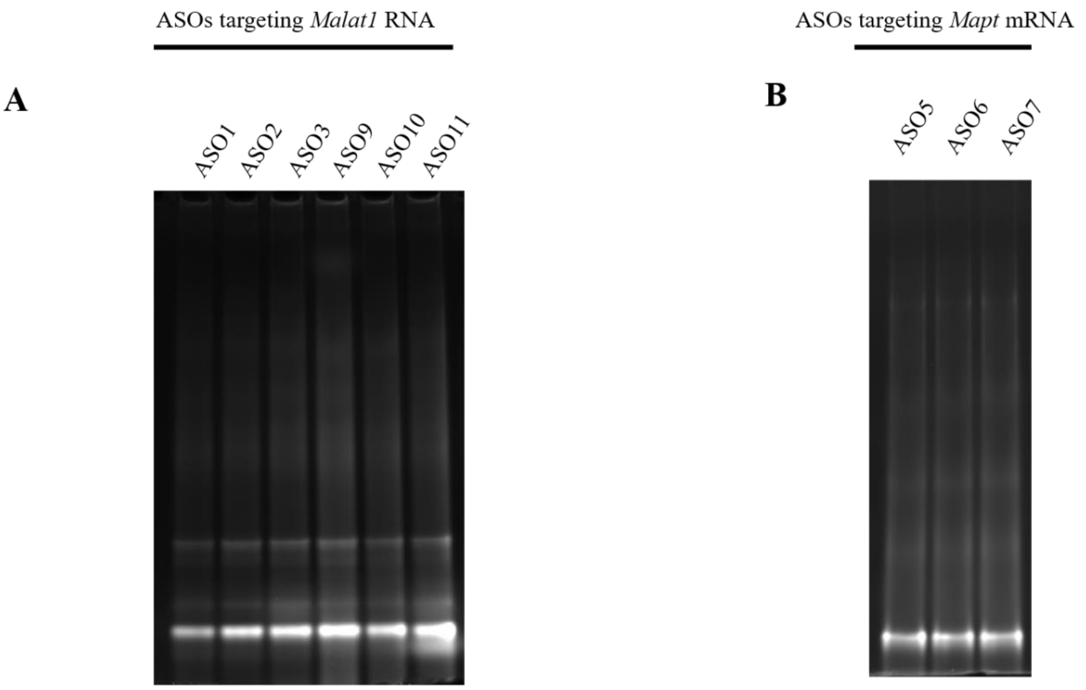

Figure S1. Electrophoretic mobility shift assay of ASOs targeting mouse *Malat1* RNA and *Mapt* mRNA.

(A) Interaction of 3 μM Cy5-labeled ASOs targeting *Malat1* RNA (ASO1, ASO2, ASO3, ASO9, ASO10, and ASO11) with proteins in human CSF. (B) Interaction of 3 μM Cy5-labeled ASOs targeting *Mapt* mRNA (ASO5, ASO6, and ASO7) with proteins in human CSF.

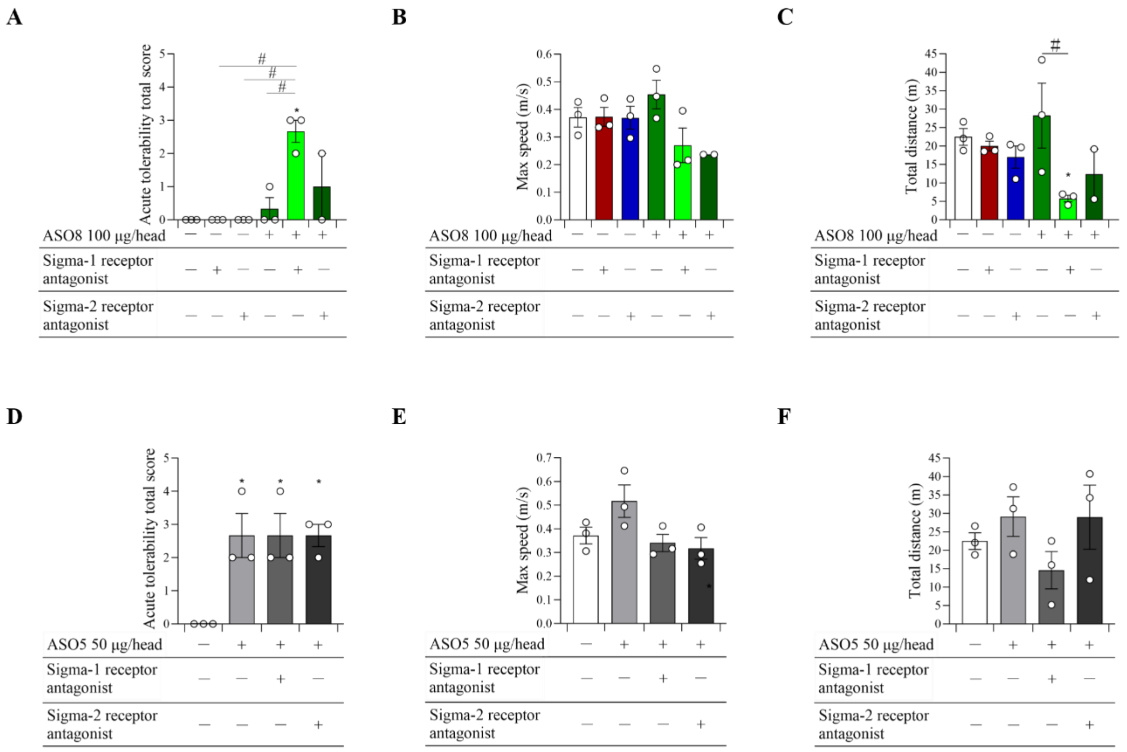

Figure S2. Assessment of acute CNS toxicity by ICV injection of 16-mer ASOs after pretreatment with the sigma-1 and -2 receptor antagonist.

(A) Acute tolerability scores in mice 1 h after the ICV injection of PBS as a negative control, 0.43 μmol sigma-1 receptor antagonist (BD 1063 dihydrochloride), 0.25 μmol sigma-2 receptor antagonist (SM21 maleate), and 19.0 nmol 16-mer ASO with 2 BNAP-AEO (ASO8; 100 μg/head) with pretreatment of PBS, sigma-1 receptor antagonist, or sigma-2 receptor antagonist. (B, C) Locomotor activity parameters, including maximum speed (B) and total distance (C), of mice shown in (A) 1 h after the ICV injection. (D) Acute tolerability scores in mice 1 h after the ICV injection of PBS and 9.5 nmol 16-mer ASO including only DNA (ASO5; 50 μg/head) with pretreatment of PBS, 0.43 μmol sigma-1 receptor antagonist, or 0.25 μmol sigma-2 receptor antagonist. (E, F) Locomotor activity parameters, including maximum speed (E) and total distance (F), of mice shown in (D) 1 h after the ICV injection.

Data are presented as mean ± standard error (n = 2-3 per group). *$p < 0.05$; data were analyzed using paired t-test with PBS. # < 0.05; data were analyzed using one-way ANOVA, followed by Tukey's post hoc test.

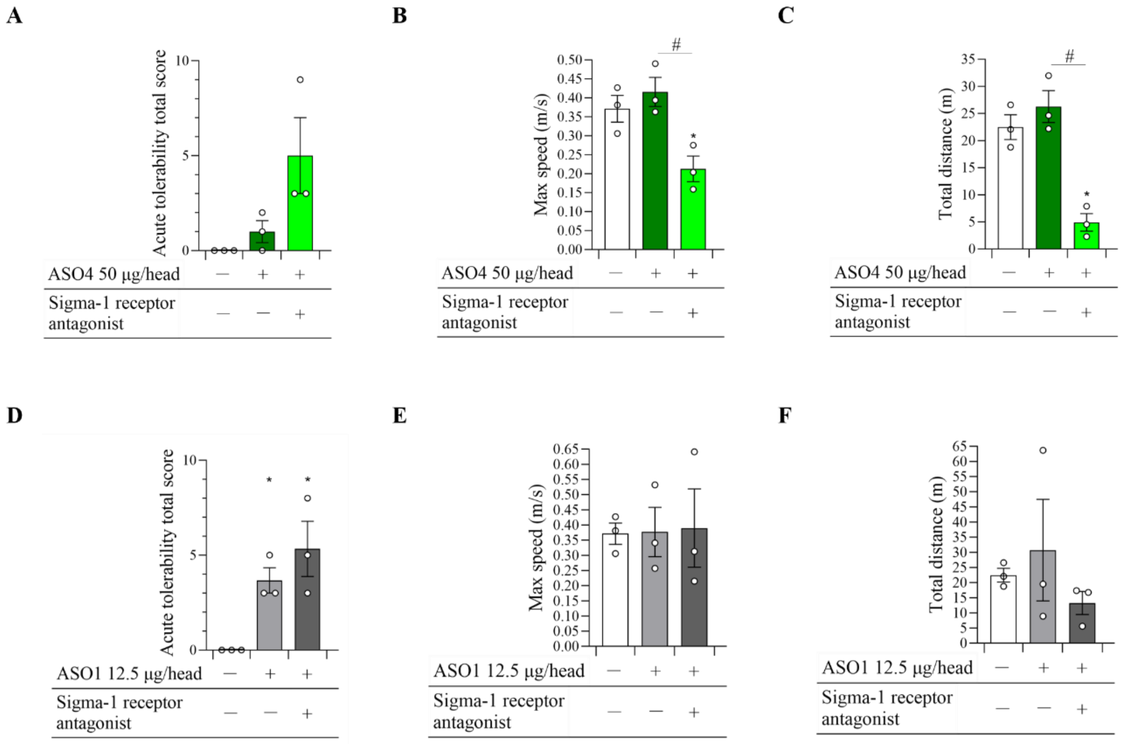

Figure S3. Assessment of acute CNS toxicity by ICV injection of the 20-mer ASO after pretreatment with the sigma-1 receptor antagonist.

(A) Acute tolerability scores in mice 1 h after the ICV injection of PBS as a negative control and 7.63 nmol 20-mer ASO with 2 BNAP-AEO (ASO4; 50 μg/head) with pretreatment of PBS or 0.43 μmol sigma-1 receptor antagonist (BD 1063 dihydrochloride). (B, C) Locomotor activity parameters, including maximum speed (B) and total distance (C), of mice shown in (A) 1 h after the ICV injection. (D) Acute tolerability scores in mice 1 h after the ICV injection of PBS and 1.91 nmol 20-mer ASO including only DNA (ASO1; 12.5 μg/head) with pretreatment of PBS or 0.43 μmol sigma-1 receptor antagonist. (E, F) Locomotor activity parameters, including maximum speed (E) and total distance (F), of mice shown in (D) 1 h after the ICV injection.

Data are presented as mean ± standard error (n = 3 per group). *p < 0.05; data were analyzed using paired t-test with PBS. # < 0.05; data were analyzed using one-way ANOVA, followed by Tukey's post hoc test.

Video S1. Mitigation of acute CNS toxicity by ICV injection of G-clamp-modified ASOs targeting *Malat1* RNA.

(A) Video of mouse was recorded 1 hour after ICV injection of PBS. (B) Video of mouse was recorded 1 hour after ICV injection of 7.63 nmol 20-mer ASO including only DNA (ASO1). (C) Video of mouse was recorded 1 hour after ICV injection of 7.63 nmol 20-mer ASO with 2 LNA (ASO2). (D) Video of mouse was recorded 1 hour after ICV injection of 7.63 nmol 20-mer ASO with 2 G-clamp (ASO3). (E) Video of mouse was recorded 1 hour after ICV injection of 7.63 nmol 20-mer ASO with 2 BNAP-AEO (ASO4).

Video S2. Mitigation of acute CNS toxicity by ICV injection of G-clamp-modified ASOs with MOE targeting *Malat1* RNA.

(A) Video of mouse was recorded 1 hour after ICV injection of 7.63 nmol 20-mer gapmer ASO with 5 and 4 MOE at the 5' and 3' ends (ASO9). (B) Video of mouse was recorded 1 hour after ICV injection of 7.63 nmol 20-mer gapmer ASO with 4 MOE and 1 LNA at the 5' end and 3 MOE and 1 LNA at the 3' end (ASO10). (C) Video of mouse was recorded 1 hour after ICV injection of 7.63 nmol 20-mer gapmer ASO with 4 MOE and 1 G-clamp at the 5' end and 3 MOE and 1 G-clamp at the 3' end (ASO11). (D) Video of mouse was recorded 1 hour after ICV injection of 7.63 nmol 20-mer gapmer ASO with 4 MOE and 1 BNAP-AEO at the 5' end and 3 MOE and 1 BNAP-AEO at the 3' end (ASO12).

Video S3. Mitigation of acute CNS toxicity by ICV injection of G-clamp-modified ASOs targeting *Mapt* mRNA.

(A) Video of mouse was recorded 1 hour after ICV injection of 19.0 nmol 16-mer ASO including only DNA (ASO5). (B) Video of mouse was recorded 1 hour after ICV injection of 19.0 nmol 16-mer ASO with 2 LNA (ASO6). (C) Video of mouse was recorded 1 hour after ICV injection of 19.0 nmol 16-mer ASO with 2 G-clamp (ASO7). (D) Video of mouse was recorded 1 hour after ICV injection of 19.0 nmol 16-mer ASO with 2 BNAP-AEO (ASO8).

Video S4. Acute CNS toxicity of mice by ICV injection of the 16-mer ASOs with presence or absence of pretreatments of the sigma receptor antagonists.

Videos were recorded at 1 hour after ICV injection of 0.43 μmol sigma-1 receptor antagonist (A), or 0.25 μmol sigma-2 receptor antagonist (B). Videos were recorded at 1 hour after ICV injection of 19.0 nmol 16-mer ASO with 2 BNAP-AEO (ASO8) with pretreatment of PBS (C), the sigma-1 receptor antagonist (D), or the sigma-2 receptor antagonist (E). Videos were recorded 1 hour after ICV injection of 9.5 nmol 16-mer ASO including only DNA (ASO5) with pretreatment of PBS (F), the sigma-1 receptor antagonist (G), or the sigma-2 receptor antagonist (H).

Video S5. Acute CNS toxicity of mice by ICV injection of the 20-mer ASOs with presence or absence of pretreatments of the sigma receptor antagonists.

Videos were recorded 1 hour after ICV injection of 7.63 nmol 20-mer ASO with 2 BNAP-AEO (ASO4) with pretreatment of PBS (A) or the sigma-1 receptor antagonist (B). Videos were recorded 1 hour after ICV injection of 1.91 nmol 20-mer ASO including only DNA (ASO1) with pretreatment of PBS (C) or the sigma-1 receptor antagonist (D).
