# Supplementary material for: Favorable efficacy and reduced acute neurotoxicity by antisense oligonucleotides with 2′,4′-BNA/LNA with 9-(aminoethoxy)phenoxazine
Source: Mol Ther Nucleic Acids. 2024 Mar 18;35(2):102161. doi: 10.1016/j.omtn.2024.102161 (PMC11229412; doi:10.1016/j.omtn.2024.102161)
Supplement: Document S2. Article plus supplemental information [file mmc27.pdf]

Molecular Therapy
Nucleic Acids
Original Article

Favorable efficacy and reduced acute neurotoxicity by antisense oligonucleotides with 2′,4′-BNA/LNA with 9-(aminoethoxy)phenoxazine

Taiki Matsubayashi,[1,4] Kotaro Yoshioka,[1,4] Su Su Lei Mon,[1,4] Maho Katsuyama,[1] Chunyan Jia,[1] Takao Yamaguchi,[2] Rintaro Iwata Hara,[1] Tetsuya Nagata,[1] Osamu Nakagawa,[2,3] Satoshi Obika,[2] and Takanori Yokota[1]

[1]Department of Neurology and Neurological Science, Graduate School of Medical and Dental Sciences, Tokyo Medical and Dental University, 1-5-45 Yushima, Bunkyo-Ku, Tokyo 113-8519, Japan; [2]Graduate School of Pharmaceutical Sciences, Osaka University, 1-6 Yamadaoka Suita, Osaka 565-0871, Japan; [3]Faculty of Pharmaceutical Sciences, Tokushima Bunri University, 180 Nishihamahoji, Yamashiro-cho, Tokushima 770-8514, Japan

An increasing number of antisense oligonucleotides (ASOs) have been approved for clinical use. However, improvements of both efficacy and safety in the central nervous system (CNS) are crucial for the treatment with CNS diseases. We aimed to overcome the crucial issues by our development of various gapmer ASOs with a novel nucleoside derivative including a 2′,4′-BNA/LNA with 9-(aminoethoxy)phenoxazine (BNAP-AEO). The various gapmer ASOs with BNAP-AEO were evaluated for thermal stability, *in vitro* and *in vivo* efficacy, and acute CNS toxicity. Thermal stability analysis of the duplexes with their complementary RNAs showed that ASOs with BNAP-AEO had a higher binding affinity than those without BNAP-AEO. *In vitro* assays, when transfected into neuroblastoma cell lines, demonstrated that ASOs with BNAP-AEO, had a more efficient gene silencing effect than those without BNAP-AEO. *In vivo* assays, involving intracerebroventricular injections into mice, revealed ASOs with BNAP-AEO potently suppressed gene expression in the brain. Surprisingly, the acute CNS toxicity in mice, as assessed through open field tests and scoring systems, was significantly lower for ASOs with BNAP-AEO than for those without BNAP-AEO. This study underscores the efficient gene-silencing effect and low acute CNS toxicity of ASOs incorporating BNAP-AEO, indicating the potential for future therapeutic applications.

INTRODUCTION

Antisense oligonucleotides (ASOs) are synthetic, short, single-stranded nucleic acids that bind to complementary RNAs in the target gene by Watson-Crick base pairing and modulate its function.[1,2] Consequently, ASOs have diverse functions, including the reduction or augmentation of gene expression, modulation of splicing, and inhibition of microRNA function, achieved through different mechanisms.[3] To enhance their binding affinity to the target RNA and resistance against nucleases, various chemical modifications, including base modifications and sugar moiety modifications such as 2′O-methoxyethyl RNA (MOE)[4] and 2′-O,4′-C-methylene-bridged nucleic acid/locked nucleic acid (2′,4′-BNA/LNA)[5,6] have been developed due to the insufficient affinity and resistance of natural oligonucleotides *in vivo*. Additionally, modification of phosphorothioate (PS) linkages from phosphodiester (PO) linkage[7] have been developed to enhance stability against nuclease-mediated degradation.

An increasing number of ASO drugs have been approved for clinical use by the U.S. Food and Drug Administration (FDA) in recent years.[8] In addition, a growing number of clinical trials have demonstrated the efficacy and safety of several ASO treatments for neurological diseases.[9–13] However, the adverse effects of ASOs, including off-target effects, immunostimulatory effects, toxicities in high-exposure organs, and thrombocytopenia,[14] have also hindered the progress of ASO drug development. Nusinersen is a fully MOE-modified ASO with PS backbone linkages, designed to modify the splicing of the targeted gene. This steric block, which is not a gapmer-type ASO, was approved for spinal muscular atrophy as the first ASO drug targeting the central nervous system (CNS). Gapmer-type ASOs, designed to silence the target gene by RNase H-mediated degradation, show promise as treatments for CNS diseases caused by gain-of-toxic function. In a phase III trial for amyotrophic lateral sclerosis, intrathecal administration of tofersen, a gapmer ASO, reduced plasma neurofilament L, a biomarker of neurodegeneration, and showed trends of decline reduction in clinical function, although it did not achieve statistically significant improvement compared with placebo.[11] CNS adverse events, including myelitis, meningitis, and lumbar radiculopathy, were also reported.[11,15] While the FDA granted accelerated approval for tofersen, the development of gapmer ASOs with high silencing efficacy and low CNS toxicity is desired for the treatment of various CNS diseases.

Received 14 September 2023; accepted 28 February 2024;
https://doi.org/10.1016/j.omtn.2024.102161.

[4]These authors contributed equally

Correspondence: Satoshi Obika, Graduate School of Pharmaceutical Sciences, Osaka University, 1-6 Yamadaoka Suita, Osaka 565-0871, Japan.
E-mail: obika@phs.osaka-u.ac.jp
Correspondence: Takanori Yokota, Department of Neurology and Neurological Science, Graduate School of Medical and Dental Sciences, Tokyo Medical and Dental University, 1-5-45 Yushima, Bunkyo-Ku, Tokyo 113-8519, Japan.
E-mail: tak-yokota.nuro@tmd.ac.jp

This is an open access article under the CC BY license (http://creativecommons.org/licenses/by/4.0/).

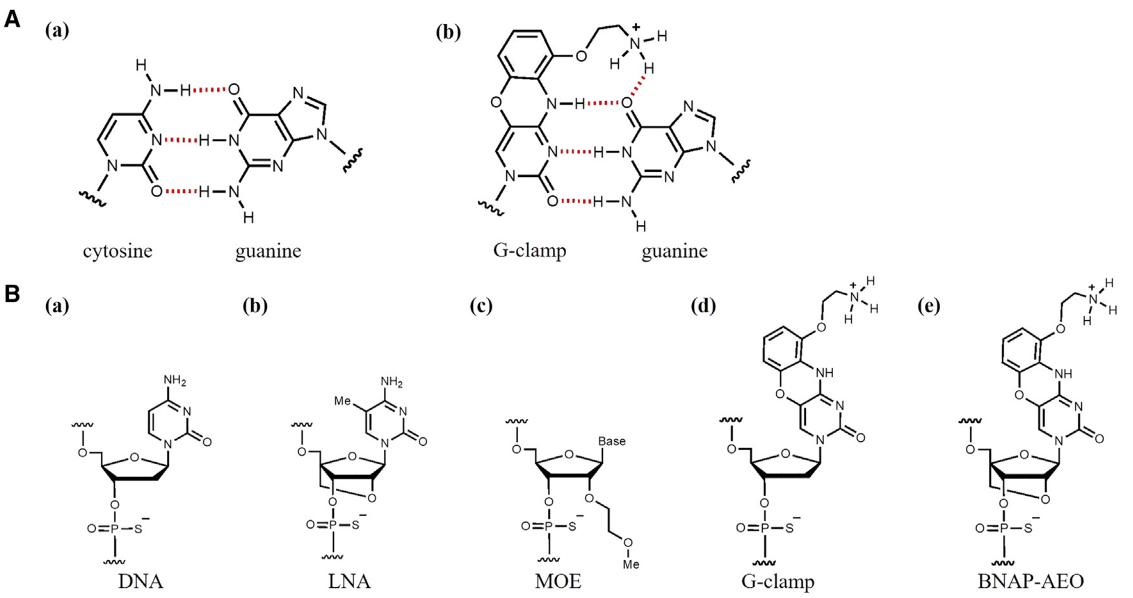

Figure 1. Base pair and structures of G-clamp and BNAP-AEO

(A) Base pairs of cytosine: guanine (a) and G-clamp: guanine (b). (B) Structures of DNA (a), LNA (b), MOE (c), G-clamp (d), BNAP-AEO (e). DNA, deoxyribonucleic acid; G-clamp, 9-(aminoethoxy)phenoxazine; BNAP-AEO, 2′,4′-BNA/LNA with a 9-(aminoethoxy)phenoxazine.

We recently developed a new chemical modification with both base- and sugar-modification called 2′,4′-BNA/LNA with a 9-(aminoethoxy)phenoxazine (BNAP-AEO).[16] The 9-(aminoethoxy) phenoxazine, also known as G-clamp, is a modified cytosine analog that forms additional hydrogen bonds with guanine, enhancing its duplex-forming abilities[17] (Figures 1A and 1B). Previous reports have shown that gapmer ASOs with G-clamp exhibited high gene silencing *in vitro* through transfection,[18] while oligonucleotides with BNAP-AEO demonstrated greater thermal stability against complementary RNAs than oligonucleotides with only G-clamp, only 2′,4′-BNA/LNA, or unmodified oligonucleotides without any chemical modification in a sugar moiety, base, and nucleotide linkage. Additionally, oligonucleotides with BNAP-AEO exhibited increased resistance to exonuclease.[16] Therefore, ASOs with BNAP-AEO are expected to have high efficacy and are promising candidates for treating CNS diseases. However, the biological efficacy and toxicity of ASOs, including BNAP-AEO, have not been investigated.

This study aimed to clarify the chemical characteristics, *in vitro* and *in vivo* efficacy, and CNS toxicity of gapmer ASOs with various chemical modifications in the base and sugar-backbone, particularly G-clamp and our novel chemical modification, "BNAP-AEO." All ASO sequences in this study were modified with full PS backbone linkages. First, we evaluated the thermal stability of duplexes formed between various ASOs with chemical modifications, including BNAP-AEO, and complementary RNA. Second, we transfected the ASOs into a neuroblastoma cell line and assessed their gene silencing effect through *in vitro* assays. Finally, we injected the ASOs into the left ventricle of mice via the intracerebroventricular (ICV) route and evaluated their gene silencing effect in the brain through *in vivo* assays. Acute CNS toxicity in mice was assessed after ICV administration of ASOs using an open field test and a tolerability scale for scoring. This study demonstrated the chemical and biological properties of gapmer ASOs with BNAP-AEO, including high thermal stability, efficient gene silencing effect in both *in vitro* and *in vivo* assays, and low acute CNS toxicity in the *in vivo* assay. All abbreviations used throughout the manuscript are summarized in Table S1.

RESULTS

ASOs with G-clamp modification have a high binding affinity to complementary RNAs and efficient gene silencing effect on *Malat1* RNA *in vitro*

We initially designed a series of PS-modified 20-mer ASOs targeting metastasis associated in lung adenocarcinoma transcript-1 (*Malat1*) RNA. The ASOs included a 20-mer ASO including only DNA ASO (ASO1), a 20-mer ASO with two LNAs (ASO2), a 20-mer ASO with two G-clamps (ASO3), and a 20-mer ASO with two BNAP-AEOs (ASO4) (Figure 2A). Regarding the design of the ASO sequences targeting *Malat1* RNA, the insertion site of G-clamp or BNAP-AEO was selected as the nearest cytosine from the 3′ or 5′ ends. The reason for the selection is because we need our next design with a limited number of MOE modifications, which were located consecutively outer side of the G-clamp or BNAP-AEO in both wing portions for exonuclease-resistance, as described in detail in the following section, to avoid too much increase in the melting temperature (T_m) value by the MOE modifications. To assess the binding affinity between the ASOs and complementary RNAs, we conducted a T_m analysis. The T_m values of 20-mer ASO with 2 G-clamp and 20-mer ASO with two LNAs were approximately 10° higher than

A

Sequence ID	ASO Sequence	T_m (°C)	IC_{50} (nM)
ASO1	gggtcagctgccaatgctag	62.9	2.0
ASO2	gggtCagctgccaatgCtag	71.0	1.85
ASO3	gggtXagctgccaatgXtag	71.1	0.62
ASO4	gggtYagctgccaatgYtag	77.6	0.82

C: LNA with 5-methylcytosine, X: G-clamp, Y: BNAP-AEO.

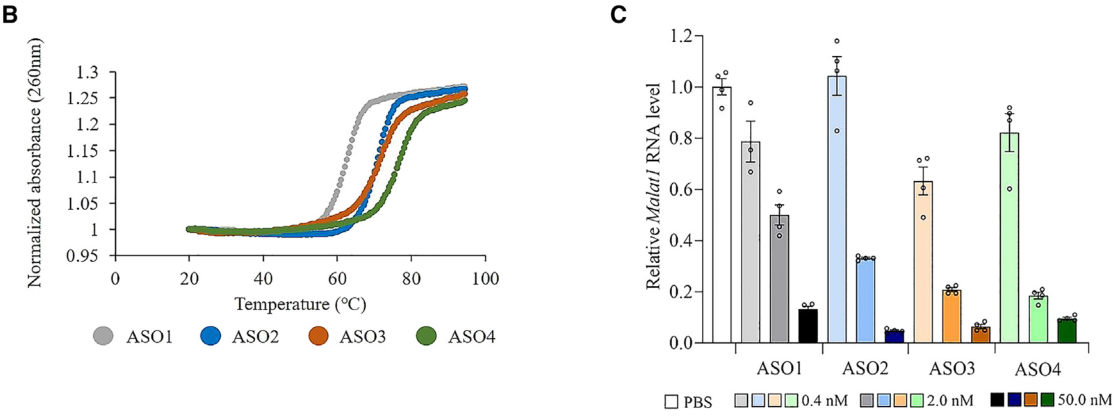

Figure 2. Increased T_m and enhanced *in vitro* inhibitory effect of ASO with G-clamp modification on *Malat1* RNA expression

(A) Structures, T_m value, and IC_{50} of 20-mer ASOs targeting mouse *Malat1* RNA: ASO1, ASO2, ASO3, and ASO4. IC_{50} values for the reduction of *Malat1* RNA expression levels in Neuro-2a mouse neuroblastoma cells 48 h after transfection. (B) The melting curve of ASO1–ASO4. UV melting profiles were measured in PBS at a scan rate of 0.5°C min^{-1} at 260 nm. The concentration of all ASOs was 2 μM. (C) Quantitative real-time PCR analysis of the relative *Malat1* RNA expression levels in Neuro-2a mouse neuroblastoma cells 48 h after transfection of 0.4, 2, and 50 nM ASO1-4. a, c, g, t: 2′-deoxyribonucleoside with adenine (a), cytosine (c), guanine (g), thymine (t). (C) 2′,4′-BNA/LNA with 5-methylcytosine. X, a 9-(aminoethoxy)phenoxazine with cytosine (G-clamp). Y, 2′,4′-BNA/LNA and 9-(aminoethoxy)phenoxazine with cytosine (BNAP-AEO). Data are presented as mean ± standard error (n = 3 or 4 per group).

20-mer ASO including only DNA (Figures 2A and 2B). The 20-mer ASO with two BNAP-AEOs exhibited the highest T_m, surpassing both 20-mer ASO with two G-clamps and 20-mer ASO with two LNAs by 6.5°. The *in vitro* potency of ASOs with G-clamp modification was evaluated by measuring expression levels of *Malat1* RNA in Neuro-2a mouse neuroblastoma cells 48 h after transfection with 0.4, 2, and 50 nM ASOs with and without G-clamp modification (Figure 2C). The gene silencing potency was further evaluated using the half-maximal inhibitory concentration (IC_{50}) value (Figure 2A). The 20-mer ASO with two G-clamps and the 20-mer ASO with two BNAP-AEOs exhibited greater gene silencing effects compared with the 20-mer ASO including only DNA and the 20-mer ASO with two LNAs, respectively. While the 20-mer ASO with two LNAs demonstrated greater gene silencing effect than the 20-mer ASO including only DNA, the 20-mer ASO with two BNAP-AEOs and the 20-mer ASO with two G-clamps showed comparable IC_{50} values.

ASOs with G-clamp modification have a high binding affinity to complementary RNAs and efficient gene silencing effect on *Mapt* mRNA *in vitro*

We next designed a series of PS-modified 16-mer ASOs targeting microtubule-associated protein tau (*Mapt*) mRNA. The ASOs included a 16-mer ASO including only DNA (ASO5), a 16-mer ASO with two LNAs (ASO6), a 16-mer ASO with two G-clamps (ASO7), and a 16-mer ASO with two BNAP-AEOs (ASO8) (Figure 3A). Regarding the design of ASO sequences targeting *Mapt* mRNA, two G-clamps or BNAP-AEOs were inserted at sites where both adjacent bases were cytosines, because our previous study showed that the effect of G-clamp or BNAP-AEO at such cytosine sites flanked by cytosine on both sides on the T_m increase is greater than those at other sites.[16] We assessed the binding affinity to complementary RNAs and the *in vitro* gene silencing effect of ASOs with G-clamp modification, compared with ASOs without G-clamp modification. The melting curves of 16-mer ASO with two G-clamps and 16-mer ASOs with two BNAP-AEOs shifted to the right compared with a 16-mer ASO, including only DNA and a 16-mer ASO with two LNAs, respectively (Figure 3B). The T_m values calculated from the melting curves were over 15° higher in the 16-mer ASO with two G-clamps and the 16-mer ASO with two BNAP-AEOs than in the 16-mer ASO including only DNA and 16-mer ASO with two LNAs, respectively. After 48 h of transfection with 1, 5, 25, and 50 nM ASOs into Neuro-2a mouse neuroblastoma cells (Figures 3A and 3C), ASOs with G-clamp modification (ASO7 and ASO8) exhibited higher inhibitory effects than ASOs without G-clamp modification (ASO5 and ASO6). The 16-mer ASO with two LNAs showed a higher gene silencing effect than 16-mer ASO including only DNA, while the gene silencing effect of 16-mer ASO with two BNAP-AEOs, calculated by IC_{50} value, was not higher than that of the 16-mer ASO with two G-clamps.

A

Sequence ID	Sequence	T_m (°C)	IC_{50} (nM)
ASO5	tagccctaaagtccca	57.0	>50
ASO6	tagcCctaaagtcCca	68.5	10.24
ASO7	tagcXctaaagtcXca	75.8	0.82
ASO8	tagcYctaaagtcYca	85.3	1.82

C: LNA with 5-methylcytosine, X: G-clamp, Y: BNAP-AEO.

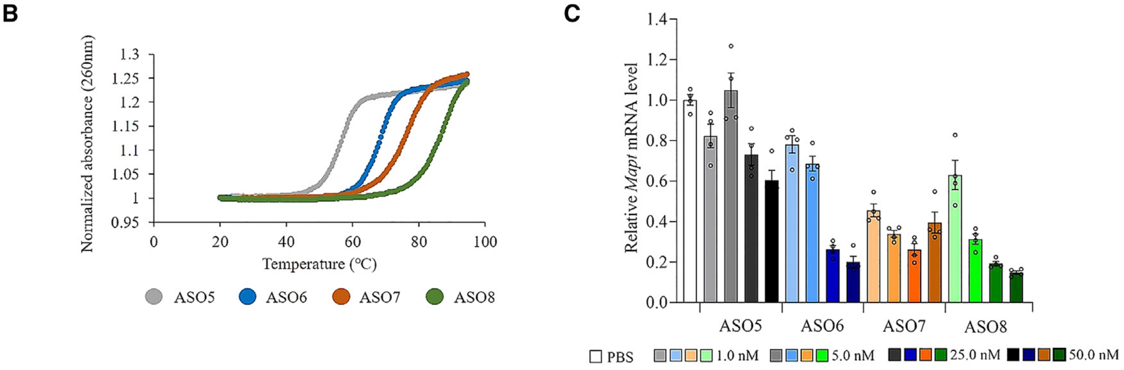

Figure 3. Increased T_m and enhanced *in vitro* inhibitory effect of ASO with G-clamp modification on *Mapt* mRNA expression

(A) Structures, T_m values, and IC_{50} of the 16-mer ASO targeting mouse *Mapt* mRNA: ASO5, ASO6, ASO7, and ASO8. IC_{50} values for the reduction of *Mapt* mRNA expression levels in Neuro-2a mouse neuroblastoma cells 48 h after transfection. (B) The melting curve of ASO5–ASO8. UV melting profiles were measured in PBS at a scan rate of 0.5°C min^{-1} at 260 nm. The concentration of all ASOs was 2 μM. (C) Quantitative real-time PCR analysis of the relative *Mapt* mRNA expression levels in Neuro-2a mouse neuroblastoma cells 48 h after transfection of 1, 5, 25, and 50 nM ASO5-8. a, c, g, t, 2′-deoxyribonucleoside with adenine (a), cytosine (c), guanine (g), thymine (t); C, 2′,4′-BNA/LNA with 5-methylcytosine; X, a 9-(aminoethoxy)phenoxazine with cytosine (G-clamp); Y, 2′,4′-BNA/LNA and 9-(aminoethoxy)phenoxazine with cytosine (BNAP-AEO). Data are presented as mean ± standard error (n = 4 per group).

MOE modified ASOs with G-clamp modification have a high binding affinity to complementary RNAs

We synthesized 20-mer ASOs targeting *Malat1* RNA with MOE modification in the wing portions of the ASOs shown in Figure 2A. The ASOs included a 20-mer gapmer ASO with five and four MOEs at the 5′ and 3′ ends (ASO9), a 20-mer gapmer ASO with four MOEs and one LNA at the 5′ end and three MOEs and one LNA at the 3′ end (ASO10), a 20-mer gapmer ASO with four MOEs and one G-clamp at the 5′ end and three MOEs and one G-clamp at the 3′ end (ASO11), and a 20-mer gapmer ASO with four MOEs and one BNAP-AEO at the 5′ end and three MOEs and one BNAP-AEO at the 3′ end (ASO12) (Figure 4A). The use of MOE modification for gapmer ASOs increases metabolic stability, enhances affinity for target mRNA, and maintains RNase H activity. This modification has been successfully applied in clinical practice with gapmer ASOs such as inotersen, volanesorsen, and tofersen.[19] We then evaluated the binding affinity to complementary RNAs and the *in vitro* gene silencing effect. The melting curves of 20-mer gapmer ASO with four MOEs and one G-clamp at the 5′ end and three MOEs and one G-clamp at the 3′ end and a 20-mer gapmer ASO with four MOEs and one BNAP-AEO at the 5′ end and three MOEs and one BNAP-AEO at the 3′ end shifted to the right compared with 20-mer gapmer ASO with five and four MOEs at the 5′ and 3′ ends and 20-mer gapmer ASO with four MOEs and one LNA at the 5′ end and three MOEs and one LNA at the 3′ end, respectively (Figure 4B). The 20-mer gapmer ASO with four MOEs and one G-clamp at the 5′ end and three MOEs and one G-clamp at the 3′ end and 20-mer gapmer ASO with four MOEs and one BNAP-AEO at the 5′ end and three MOEs and one BNAP-AEO at the 3′ end exhibited approximately 6° higher T_ms than the 20-mer gapmer ASO with five and four MOEs at the 5′ and 3′ ends and the 20-mer gapmer ASO with four MOEs and one LNA at the 5′ end and three MOEs and one LNA at the 3′ end, respectively (Figure 4A). After 48 h of transfection with 0.4, 2, 10, and 50 nM ASOs into Neuro-2a mouse neuroblastoma cells (Figures 4A and 4C), 20-mer gapmer ASO with four MOEs and one BNAP-AEO at the 5′ end and three MOEs and one BNAP-AEO at the 3′ end demonstrated similar knockdown efficacy as the other MOE-gapmers (ASO9, ASO10, and ASO11).

ASOs with G-clamp modification targeting *Malat1* RNA potently suppress gene expression *in vivo*

ASOs with G-clamp modification demonstrated high binding affinity to complementary RNAs and efficient knockdown efficacy *in vitro*. We then proceeded to evaluate the gene silencing effect of ASOs with G-clamp modification on *Malat1* RNA *in vivo* (Figures 2A and 4A). For this experiment, we injected 7.63 nM/head (equivalent to 50 μg/head) into the left ventricle of mice via the ICV route and assessed the gene silencing effect in various areas of the brain (hippocampus, occipital cortex, and frontal cortex) 7 days after ICV administration (Figures 5A–5C). All the ASOs used in this experiment had

A

Sequence ID	Sequence	T_m (°C)	IC_{50} (nM)
ASO9	GGGTCagctgccaatgCTAG	71.9	0.45
ASO10	GGGTCagctgccaatgCTAG	78.3	0.41
ASO11	GGGTXagctgccaatgXTAG	78.5	0.67
ASO12	GGGTYagctgccaatgYTAG	84.7	0.46

C: LNA with 5-methylcytosine, X: G-clamp, Y: BNAP-AEO, *A*: MOE with adenine,
C: MOE with 5-methylcytosine, *G*: MOE with guanine, *T*: MOE with thymine.

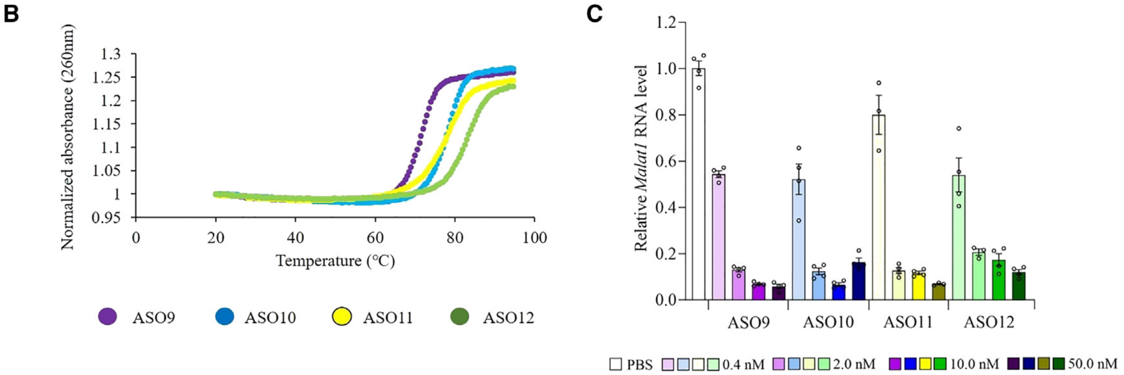

Figure 4. Increased T_m and efficient *in vitro* inhibitory effect of ASO with G-clamp modification and MOE on *Malat1* RNA expression

(A) Structures, T_m values, and IC_{50} of MOE-modified 20-mer ASOs targeting mouse *Malat1* RNA: ASO9, ASO10, ASO11, and ASO12. IC_{50} values for the reduction of *Malat1* RNA expression levels in Neuro-2a mouse neuroblastoma cells 48 h after transfection. (B) The melting curve of ASO9–ASO12. UV melting profiles were measured in PBS at a scan rate of 0.5°C min^{-1} at 260 nm. The concentration of all ASOs was 2 μM. (C) Quantitative real-time PCR analysis of the relative *Malat1* RNA expression levels in Neuro-2a mouse neuroblastoma cells 48 h after transfection of 0.4, 2, 10, and 50 nM ASO9-12. a, c, g, t, 2′-deoxyribonucleoside with adenine (a), cytosine (c), guanine (g), thymine (t); C, 2′,4′-BNA/LNA with 5-methylcytosine; *A*, *C*, *G*, *T*, MOE with adenine (*A*), 5-methylcytosine (*C*), guanine (*G*), thymine (*T*); X, a 9-(aminoethoxy)phenoxazine with cytosine (G-clamp); Y, BNAP-AEO. Data are presented as mean ± standard error (n = 3 or 4 per group).

been previously evaluated. The knockdown efficacy of 20-mer ASO with two G-clamps (ASO3) showed a tendency to be higher than that of 20-mer ASO including only DNA (ASO1), although no significant differences were observed between these groups. Additionally, *Malat1* RNA expression was more effectively suppressed by 20-mer ASO with two BNAP-AEOs (ASO4) than by 20-mer ASO with two LNAs (ASO2) in the hippocampus and occipital cortex, and a similar trend was observed in the frontal cortex. Alternatively, ASOs with MOE showed a similar gene silencing effect (ASO9-12). There were no significant differences in the gene silencing effect between ASOs with G-clamp (ASO3 and ASO11) and ASOs with BNAP-AEO (ASO4 and ASO12).

G-clamp modification in ASO targeting *Malat1* RNA mitigates acute CNS toxicity of ASO

To investigate the effect of G-clamp modification in ASOs targeting *Malat1* RNA on acute CNS toxicity *in vivo*, the neurobehaviors of mice were evaluated using the acute toxicity scoring system and open field test after ICV administration of 7.63 nM/head (equivalent to 50 μg/head) ASOs. The sequences and results of this evaluation are shown in Figures 2A and 6A–6D, respectively. The 20-mer ASO including only DNA (ASO1) and the 20-mer ASO with two LNAs (ASO2) induced high acute CNS toxicity in the acute toxicity scoring system compared with PBS (Video S1), and this toxicity persisted for 4 h after ICV administration. Furthermore, in the open field test conducted 1 h after ICV administration, mice injected with the 20-mer ASO including only DNA and 20-mer ASO with two LNAs showed significantly lower maximum speed, total distance, and mobile time compared with the PBS group. Surprisingly, the acute CNS toxicity in mice injected with the 20-mer ASO with two G-clamps (ASO3) and the 20-mer ASO with two BNAP-AEOs (ASO4) was dramatically reduced compared with mice injected with the 20-mer ASO, including only DNA, and the 20-mer ASO with two LNAs, respectively (Video S1), although injection of the 20-mer ASO with two G-clamps and the 20-mer ASO with two BNAP-AEOs also resulted in mild acute CNS toxicity, which lasted up to 2 h after ICV administration in the acute toxicity scoring system and was detected as lower locomotor activity relative to PBS injection in the open field test conducted 1 h after injection. We further evaluated the acute CNS toxicity of MOE-modified ASOs. The sequences and results of this evaluation are shown in Figures 4A and 6E–6H, respectively. In mice injected with a 20-mer gapmer ASO with five and four MOEs at the 5′ and 3′ ends (ASO9) and a 20-mer gapmer ASO with four MOEs and one LNA at the 5′ end and three MOEs and one LNA at the 3′ end (ASO10), acute CNS toxicity and significantly lower locomotor activity than mice injected with PBS were observed in the acute toxicity scoring system and open field test, respectively (Video S2). Both the ASOs including G-clamp (ASO11) or BNAP-AEO (ASO12) modifications with MOE-modified wings showed no neurotoxicity in the assessment with the acute toxicity scoring system and also almost no abnormality in the open-field test (Video S2), decreasing the toxicity significantly compared with the ASO including LNA modification in the MOE

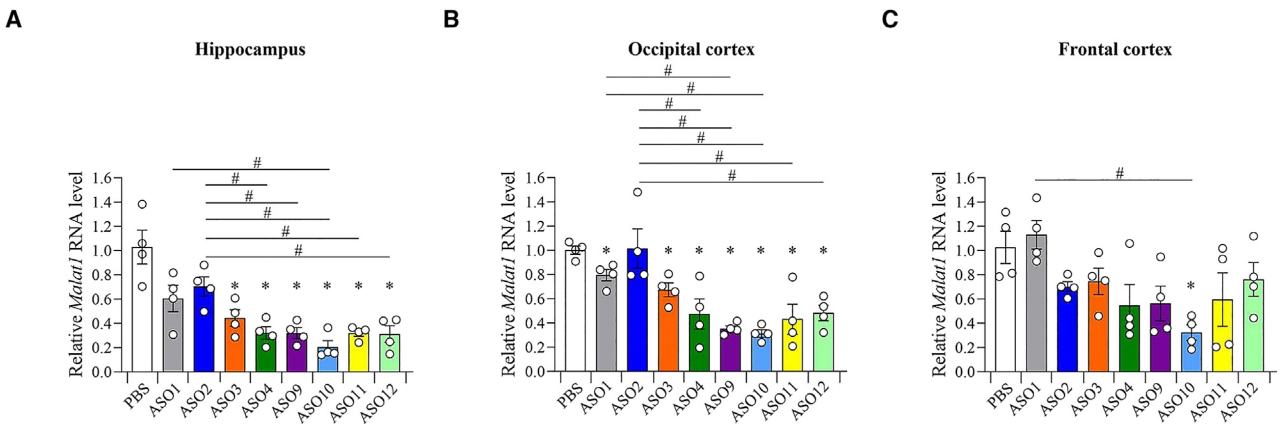

Figure 5. Efficient *in vivo* inhibitory effect of ASO with G-clamp modification on *Malat1* RNA expression
Quantitative real-time PCR analysis of the relative *Malat1* RNA expression levels in the hippocampus (A), occipital cortex (B), and frontal cortex (C) of the mouse brain 7 days after the ICV injection of ASO1–ASO4 and ASO9–ASO12 at 7.63 nM/head. Data are presented as mean ± standard error (n = 4 per group). *$p < 0.05$; data were analyzed using paired t-test with PBS. #$p < 0.05$; data were analyzed using one-way ANOVA, followed by Tukey's post hoc test.

wings (ASO10), as well as ASO with only MOE-modified wings (ASO9). Importantly, acute CNS toxicity was not observed in mice injected with the 20-mer gapmer ASO with four MOEs and one G-clamp at the 5′ end and three MOEs and one G-clamp at the 3′ end and the 20-mer gapmer ASO with four MOEs and one BNAP-AEO at the 5′ end and three MOEs and one BNAP-AEO at the 3′ end, and there were no significant differences in locomotor activity in the open-field test conducted 1 h after ICV administration between these mice and PBS-injected mice. These findings indicate that G-clamp modification mitigates acute CNS toxicity in gapmer ASOs, and the combination of G-clamp and MOE has a beneficial effect on decreasing toxicity.

ASOs with G-clamp modification targeting *Mapt* mRNA potently suppress gene expression *in vivo*

We evaluated the gene silencing effect in various areas of the mouse brain (hippocampus, occipital cortex, and frontal cortex) 7 days after ICV administration of 19.0 nM/head (equivalent to 100 μg/head) ASOs targeting *Mapt* mRNA (Figure 3A). The 16-mer ASO with two G-clamps (ASO7) exhibited significantly better inhibition effects than the 16-mer ASO including only DNA (ASO5) in the hippocampus, occipital cortex, and frontal cortex. In contrast, the suppressive effect of the 16-mer ASO with two BNAP-AEOs (ASO8) on *Mapt* mRNA levels was comparable with that of the 16-mer ASO with two LNAs (ASO6) (Figures 7A–7C). The knockdown efficacy of the 16-mer ASO with two BNAP-AEOs was almost equivalent to that of the 16-mer ASO with two G-clamps.

G-clamp modification in ASO targeting *Mapt* mRNA mitigates the acute CNS toxicity of ASOs

The effect of G-clamp modification in ASO targeting *Mapt* mRNA on acute CNS toxicity *in vivo* was evaluated as neurobehaviors of mice in an acute toxicity scoring system and open-field test after ICV administration of 19.0 nM/head (equivalent to 100 μg/head) ASOs. The ASO sequences and test results are shown in Figures 3A and 8A–8D, respectively. The injection of 16-mer ASO including only DNA (ASO5) and 16-mer ASO with two LNAs (ASO6) led to high acute CNS toxicity in both the acute toxicity scoring system and open-field test (Video S3). In the acute toxicity scoring system and open-field test, the mice injected with the 16-mer ASO with two G-clamps (ASO7) and the 16-mer ASO with two BNAP-AEOs (ASO8) had lower abnormal neurobehavior compared with mice injected with the 16-mer ASO, including only DNA and 16-mer ASO with two LNAs, respectively (Video S3).

G-clamp modification does not alter the binding properties to proteins in cerebrospinal fluid

While protein binding is mainly influenced by PS linkages,[20] a previous study elucidated the variations in protein binding to gapmer ASOs with PS backbone linkages, highlighting differences arising from chemical modifications between 2′,4′-BNA/LNA and MOE.[21] To determine whether the difference in acute neurotoxicity in ASOs with different chemical modifications depended on the binding properties to proteins in cerebrospinal fluid (CSF), electrophoretic mobility shift assay with ASOs and human CSF was performed. Similar protein-binding properties of ASOs were detected, whether or not G-clamp modification was introduced in ASOs targeting *Malat1* RNA (Figure S1A) and *Mapt* mRNA (Figure S1B).

Pretreatment of sigma-1 receptor antagonist induces CNS toxicity with the ASOs including BNAP-AEO more significantly compared with the ASOs without the modification

The mechanism underlying acute CNS toxicity caused by ASOs is not fully understood, but recent papers reported that the neurotoxicity of mice injected with ASOs is associated with intracellular calcium homeostasis in neurons.[22,23] In addition, modulation of cellular calcium responses is subject to the sigma-1 receptor expressed abundantly in the brain,[24,25] including neurons.[26] Moreover, several papers showed that ligands against the sigma-1 receptor exhibit a characteristic structural pattern as aminoalkyl group attached to a hydrophobic aromatic ring and the G-clamp modification poses a similar structure to

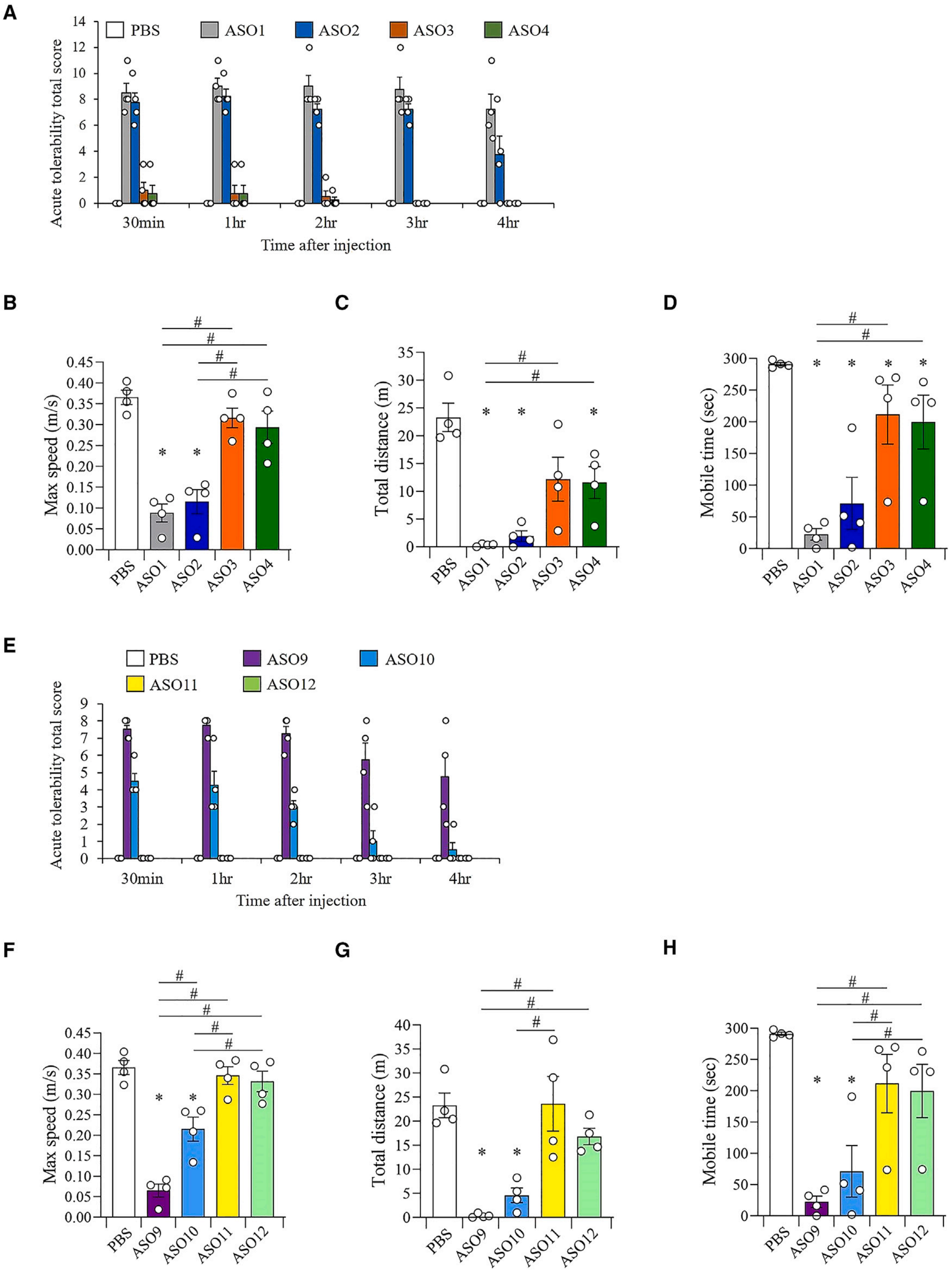

Figure 6. Mitigation of acute CNS toxicity by ICV injection of G-clamp-modified ASOs targeting *Malat1* RNA

(A) Acute tolerability scores in mice 0.5, 1, 2, 3, and 4 h after the ICV injection of 20-mer ASOs targeting mouse *Malat1* RNA at 7.63 nM/head: ASO1, ASO2, ASO3, and ASO4. (B–D) Locomotor activity parameters, including maximum speed (B), total distance (C), and mobile time (D), of mice 1 h after the ICV injection of ASO1–ASO4. (E) Acute

(legend continued on next page)

the groups.[27–29] We then hypothesized that the G-clamp modifications in the ASOs have a capability to interact with the sigma-1 receptor and modulate calcium levels in neurons, leading to the improvements of the neurotoxicity in mice injected with the ASOs.

To investigate the effect of modulators for the sigma-1 receptor on neurotoxicity in the ASOs, we assessed acute CNS toxicity in mice after ICV administration of the ASOs with pretreatment of antagonist against two types of sigma receptor, BD 1063 dihydrochloride for the sigma-1 receptor and SM21 maleate for the sigma-2 receptor.[26,30] Initially, we preliminarily determined the maximum tolerable dose, i.e., 0.43 μM for BD 1063 dihydrochloride and 0.25 μM for SM21 maleate, where neither compound induced neurotoxicity of mice through scoring with the acute toxicity scoring system or open-field tests. Next, we administered 19.0 nM/head (equivalent to 100 μg/head) of the 16-mer ASO with 2 BNAP-AEO (ASO8) or 9.5 nM/head (equivalent to 50 μg/head) of the 16-mer ASO, including only DNA (ASO5) into mice with or without the two antagonists, respectively. As shown in Figures S2A–S2C and Video S4, mice with the pretreatment of the sigma-1 antagonist showed significantly higher CNS toxicity with ASO8 compared with mice without the antagonist in the acute toxicity scoring system, as well as the open field test. In contrast, a significant difference in the neurotoxicity of mice injected with ASO8 was not observed between the groups with or without the sigma-2 receptor antagonist. In contrast, mice injected with ASO5 (Figures S2D–S4F) did not show significant differences in CNS toxicity between groups with the presence or absence of pretreatments of the sigma-1 receptor antagonist, as well as the sigma-2 receptor antagonist.

We conducted further assessments to evaluate the effect of the sigma-1 receptor on neurotoxicity in the ASOs with other sequences. Analysis of acute CNS toxicity in mice injected with 7.63 nM/head (equivalent to 50 μg/head) of 20-mer ASO with two BNAP-AEOs (ASO4) or 1.91 nM/head (equivalent to 12.5 μg/head) of 20-mer ASO including only DNA (ASO1) found that pretreatment of the sigma-1 receptor antagonist before ASO4 injection induced significantly greater acute CNS toxicity compared with no pretreatment (Figures S3A–S3C and Video S5); in contrast, the pretreatment of the antagonist before ASO1 injection did not (Figures S3D–S3F).

These findings indicate that the sigma-1 receptor antagonist has a more significant effect on the neurotoxicity of the ASO with BNAP-AEO modification compared with that of the ASO without that modification, consistent with our hypothesis that the BNAP-AEO modification decreases the ASO neurotoxicity through its interaction with the sigma-1 receptor.

DISCUSSION

In this study, we synthesized various gapmer ASOs, including BNAP-AEO or its derivative, and evaluated their binding affinity to complementary RNAs, gene silencing effects *in vitro* and *in vivo*, and acute CNS toxicity. Our results revealed three notable findings related to ASOs with BNAP-AEO. First, ASOs with BNAP-AEO modification exhibited a greater binding affinity for complementary RNAs compared with ASOs without BNAP-AEO modification. Second, ASOs with BNAP-AEO modification demonstrated efficient gene silencing effects on the target gene in both *in vitro* and *in vivo* assays. Finally, upon injection into mice via the ICV route, the acute CNS toxicity of the ASOs with BNAP-AEO modification, which was assessed through open-field tests and scoring systems, was significantly lower than those of the ASOs without BNAP-AEO modification. This unexpected favorable effect highlights the potential of BNAP-AEO as a chemical modification for ASOs.

The primary purpose of introducing chemical modifications into ASOs is to improve duplex stability. For example, 2′,4′-BNA/LNA induces conformational changes in DNA-RNA duplexes and prevents RNase H cleavage of the target RNA,[31] while MOE increases affinity for complementary RNAs and is highly resistant to degradation by nucleases.[4] In our study, the introduction of 2′,4′-BNA/LNA and MOE into gapmer ASOs resulted in an increase in T_m of approximately 4°C–5°C per 2′,4′-BNA/LNA and approximately 1°C per MOE, respectively. In contrast, G-clamp, a cytosine analog, was designed to improve π-π stacking interactions with adjacent nucleobases by forming additional hydrogen bonds with guanine. The previous report has shown that G-clamp modification improves the T_m of ASOs with G-clamp.[18] In our study, the introduction of G-clamp modification into gapmer ASOs (ASO1 vs. ASO3, ASO5 vs. ASO7, ASO2 vs. ASO4, ASO6 vs. ASO8, and ASO10 vs. ASO12) resulted in an increased T_m of approximately 3°C–9°C per G-clamp modification. The range of ΔT_m varied depending on the sequence, with a higher ΔT_m observed when the adjacent bases at the G-clamp insertion site were cytosine, consistent with previous reports.[16,32] Our previous study found that BNAP-AEO modification significantly increased the T_m of oligonucleotides.[16] Our current study provides further evidence of the higher ΔT_m achieved by BNAP-AEO modification in gapmer ASOs compared with other modifications, including G-clamp, 2′,4′-BNA/LNA, and MOE.

The therapeutic efficacy of ASOs is associated with their T_m against complementary RNAs.[33,34] In our study, ASOs with G-clamp modification showed a tendency for higher knockdown efficacy compared with ASOs, including DNA only, both *in vitro* and *in vivo* assays (ASO1 vs. ASO3 and ASO5 vs. ASO7). This can be attributed to the elevated T_m. However, the results of this study revealed several exceptions to the hypothesis that the potency of ASOs increases with increasing T_m values. The 20-mer gapmer ASO with four MOEs and one BNAP-AEO at the 5′ end and three MOEs and one BNAP-AEO at the 3′ end (ASO12) did not improve potency compared with other

tolerability scores in mice 0.5, 1, 2, 3, and 4 h after the ICV injection of 20-mer ASOs with MOE targeting mouse *Malat1* RNA at 7.63 nM/head: ASO9, ASO10, ASO11, and ASO12. (F–H) Locomotor activity parameters, including maximum speed (F), total distance (G), and mobile time (H), of mice 1 h after the ICV injection of ASO9–ASO12. Data are presented as mean ± standard error (n = 4 per group). *$p < 0.05$; data were analyzed using paired t-test with PBS. #$p < 0.05$; data were analyzed using one-way ANOVA, followed by Tukey's post hoc test.

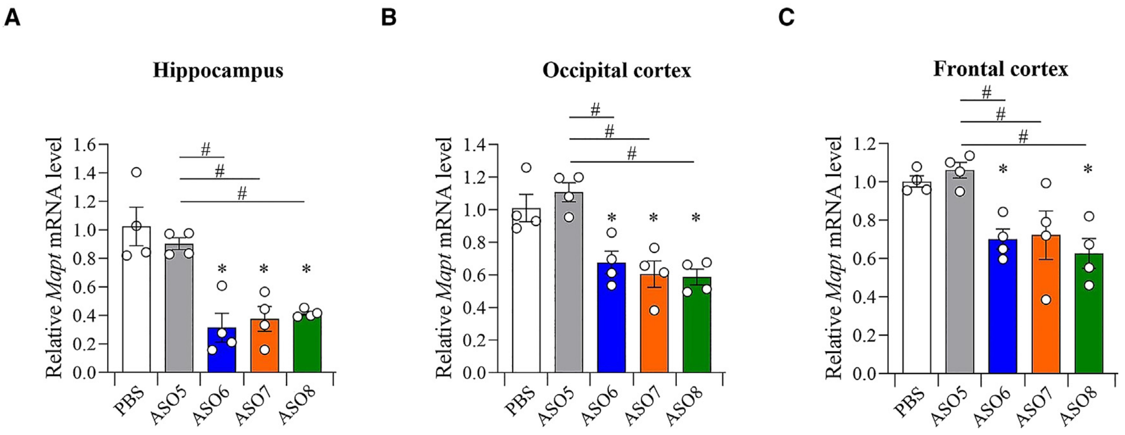

Figure 7. Efficient *in vivo* inhibitory effect of ASO with G-clamp modification on *Mapt* mRNA expression

Quantitative real-time PCR analysis of the relative *Mapt* mRNA expression levels in the hippocampus (A), occipital cortex (B), and frontal cortex (C) of the mouse brain 7 days after the ICV injection of ASO5–ASO8 at 19 nM/head. Data are presented as mean ± standard error (n = 4 per group). *$p < 0.05$; data were analyzed using paired t test with PBS. #$p < 0.05$; data were analyzed using one-way ANOVA, followed by Tukey's post hoc test.

MOE-gapmers (ASO9, ASO10, and ASO11), despite the increase in T_m. Furthermore, the gene silencing effect of ASOs with BNAP-AEO modification and ASOs with G-clamp modification was comparable in both *in vitro* and *in vivo* assays (ASO3 vs. ASO4, ASO7 vs. ASO8, and ASO11 vs. ASO12). Interestingly, the 16-mer ASO with two BNAP-AEOs (ASO8) showed a higher T_m value but a larger IC_{50} than the 16-mer ASO with two G-clamps (ASO7). These findings suggest that the knockdown activity of ASOs may not be enhanced beyond a certain T_m threshold, especially above 72°C under the experimental conditions in our study. Previous studies have shown that there is an optimal T_m range for achieving high efficacy in ASOs with LNA[35] and ASOs with G-clamps.[18] Additionally, previous studies reported that too high affinity may decrease the potency of gapmer ASOs, resulting in a J-curve or U-curve correlation between T_m values and the IC_{50}.[35,36] ASOs with excessively high T_ms may not be able to reuse the antisense sequence after RNase H cleavage or may lead to undesired binding, resulting in a low ASO concentration at the target site. ASOs with excessively high T_ms have been demonstrated to exhibit low turnover ability in RNase H-mediated silencing of the target RNA, thereby limiting the recycling of ASOs in cells.[37] The reduced ASO recycling capability due to excessively elevated T_m values may explain the reason for the reduced potency of ASO8 compared with ASO7. Although the IC_{50} of the 20-mer ASO with two LNAs (ASO2) was higher than that with 2 G-clamp (ASO3) despite similar T_m values (Figure 2A), this finding could not be explained only by the trend of the T_m values. Such an unexpected decrease in the efficacy with LNA modification compared with G-clamp was also observed in the previous paper.[18] These discrepancies between T_m and the IC_{50} may be explained by other effects of the chemical modification on other factors, such as different profiles of protein binding affecting subcellular localization.[21,38] Further verification of the detailed differences in protein binding due to the chemical modifications of LNA and G-clamp, as well as their intracellular dynamics, will be required in the future. Future studies should also focus on designing BNAP-AEO-modified ASOs with an optimal T_m by adjusting the sequence structure, as BNAP-AEO modification has the potential to significantly increase T_m in ASOs.

The mechanism underlying the acute CNS toxicity caused by ASOs is not fully understood, but our previous study suggested that decreased intracellular free calcium levels induced by ASOs may play a role in acute CNS toxicity in mice.[22] Additionally, Hagedorn et al.[23] reported that base differences in ASOs, such as the number of guanine nucleotides (which are highly neurotoxic) or the distance between the 3′ end and the nearest guanine nucleotides, influenced calcium oscillations in cultured neurons, thus predicting neurotoxicity in mice. Furthermore, the chemical modification of ASOs can also affect acute CNS toxicity. In our previous study, ASOs with MOE modification and PO linkage showed lower acute CNS toxicity compared with those with 2′,4′-BNA/LNA and PS linkage, respectively.[22] This difference may be attributed to variations in protein-binding properties, as ASOs with MOE modifications and PO linkage have a weaker binding affinity for proteins compared with 2′,4′-BNA/LNA and PS linkage.[21,39,40] In this study, the introduction of a G-clamp modification into ASOs dramatically decreased acute neurotoxicity. This decrease may be due to differences in changes in intracellular calcium levels and/or protein binding after ICV injection between ASOs with G-clamp and ASOs with other chemical modifications. Notably, G-clamp structurally resembles sigma-1 receptor agonists.[27–29] *In vitro* studies have shown that sigma-1 receptors are rapidly upregulated under cellular stress and play a role in regulating intracellular calcium homeostasis.[24,25] Agonists of the sigma-1 receptor have been shown to potentiate increases in intracellular free calcium levels associated with the activation of inositol 1,4,5-trisphosphate receptors, while antagonists reduce this calcium signal.[41] In addition, agonists of the sigma-1 receptor have been reported to mitigate increases in intracellular free calcium levels by inhibiting N-methyl-D-aspartate receptors, and antagonists caused a reaction in the opposite

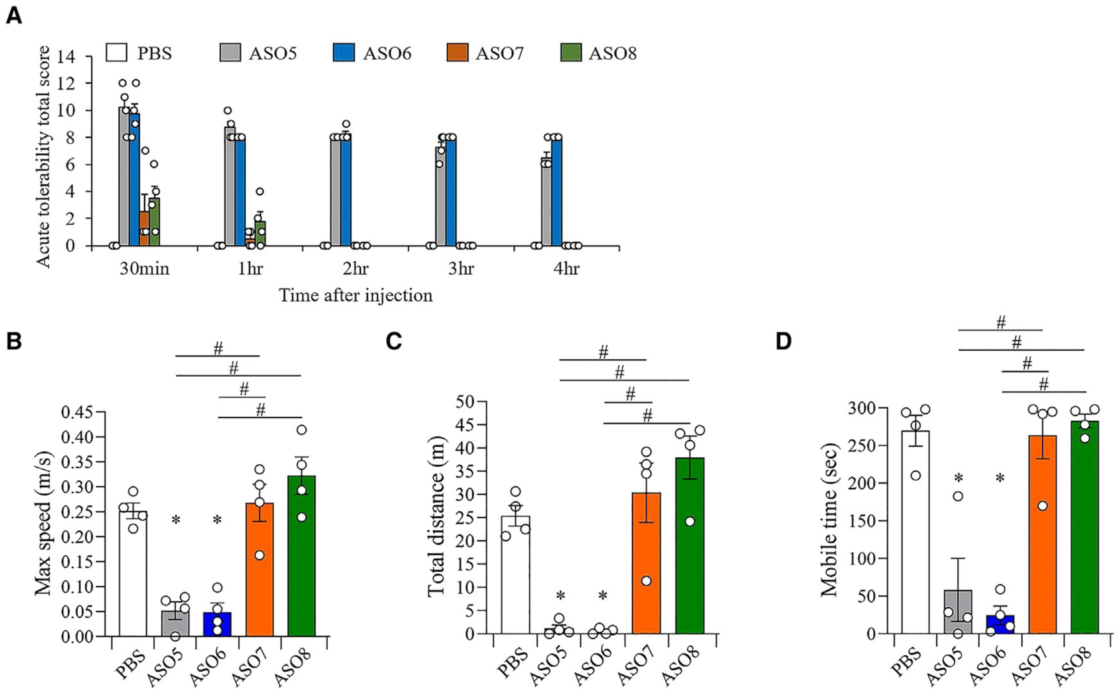

Figure 8. Mitigation of acute CNS toxicity by ICV injection of G-clamp-modified ASOs targeting *Mapt* mRNA

(A) Acute tolerability scores in mice 0.5, 1, 2, 3, and 4 h after the ICV injection of 16-mer ASO targeting mouse *Mapt* mRNA at 19.0 nM/head: ASO5, ASO6, ASO7, and ASO8. (B–D) Locomotor activity parameters, including maximum speed (B), total distance (C), and mobile time (D), of mice 1 h after the ICV injection of ASO5–ASO8. Data are presented as mean ± standard error (n = 4 per group). *p < 0.05; data were analyzed using paired t test with PBS. #p < 0.05; data were analyzed using one-way ANOVA, followed by Tukey's post hoc test.

direction.[42] Therefore, G-clamp may function as an agonist of the sigma-1 receptor, mitigating the reduction of intracellular free calcium levels caused by ASOs and contributing to the low neurotoxicity observed in ASOs with G-clamp modification. Electrophoretic mobility shift assay results in this study indicated that binding properties to extracellular proteins in CSF did not affect the acute CNS toxicity of ASOs. However, this may be due to the stronger correlation between neurotoxicity and cell-surface or intracellular proteins, including the sigma-1 receptor. In our study, the pretreatment with the sigma-1 receptor antagonist induced acute CNS toxicity more significantly with the ASOs with G-clamp modification than with those without G-clamp modification. Additionally, the sigma-1 receptor antagonist had greater effects on the neurotoxicity of ASO with G-clamp modification than the sigma-2 receptor antagonist, another subtype in sigma receptors. These findings support our hypothesis that ASOs with G-clamp modification ameliorate acute CNS toxicity by acting as an agonist of the sigma-1 receptor. Importantly, ASOs with G-clamp modifications exhibited less acute CNS toxicity compared with those with MOE modifications, which are also used in nusinersen, a fully PS-MOE-modified splice-modulating ASO that is not of the gapmer type, due to their high safety profile.[43] Therefore, G-clamp modification holds promise for future clinical applications. Furthermore, while guanine is known to be a highly toxic base, the introduction of G-clamp, a cytosine analog, into ASOs reduced acute neurotoxicity in this study. This finding suggests that alternatives to cytosine could serve as a new strategy to decrease ASO toxicity.

One of the side effects of ASO administration is cytotoxicity caused by the activation of innate immunity.[14] Previous reports demonstrated that ASOs administered via the ICV route could lead to the activation of astroglial or microglial responses, inducing inflammation in the brain.[44,45] Importantly, innate immunity caused by ASOs modified with full PS backbone linkages varies dramatically, depending on the type of chemical modification.[46] Because the detailed mechanism of innate immunity immediately after ICV ASO administration remains unknown, further studies are necessary to investigate the correlation between acute CNS toxicity and innate immunity after ICV administration of ASO.

The *in vitro* results of this study show that all ASOs, except the 16-mer ASO including only DNA (ASO5), exhibited a dose-dependent gene silencing effect up to a certain concentration. Notably, ASOs with BNAP-AEO modification also had the dose-dependent gene silencing effect, marking a potentially valuable aspect for future clinical applications. Furthermore, the inhibitory effect on *Malat1* RNA increased up to 50 nM with approximately 90% knockdown efficacy for 20-mer ASOs without MOE modification (ASO1–ASO4), while potency was saturated above 2 nM with comparable knockdown efficacy for MOE-modified 20-mer ASOs (ASO9–ASO12), suggesting a notably high efficacy of MOE-modified ASOs.

A few limitations were present in this study. First, G-clamp is an analog of cytosine, which limited the sequence selection of ASO. Second, further research is needed in the future to clarify the mechanism by which G-clamp reduces acute CNS toxicity.

ASOs, including BNAP-AEO, were first synthesized in this study. Our results demonstrated the favorable effects of BNAP-AEO modification on ASOs: high binding affinity to complementary RNAs, efficient gene suppression, and low acute CNS toxicity. These features of BNAP-AEO might overcome the trade-offs between efficacy and neurotoxicity in ASOs with chemical modifications, such as PS linkage and 2′,4′-BNA/LNA. Therefore, we believe that BNAP-AEO could promote the clinical application of ASOs targeting CNS diseases.

MATERIALS AND METHODS

Design and synthesis of ASOs

The sequence and design of chemical modifications in the ASO used in this study were derived from previous studies showing 20-mer ASOs targeting *Malat1* RNA[22,47] and 16-mer ASOs targeting *Mapt* mRNA.[23] These original ASOs were also reported to have both gene-silencing effects and acute CNS toxicity in mice.[22,23] In this study, the design of MOE-modified 20-mer ASOs originated from a 5-10-5 MOE-modified gapmer because this design is one of the possible ASOs for the clinical practice of treatment for neurological diseases. For example, tofersen with the same design was approved by the FDA for amyotrophic lateral sclerosis associated with the *SOD-1* gene.[48] The sequence and design of the 16-mer ASOs targeting *Mapt* mRNA originated from a 3-10-3 LNA-modified gapmer ASO in a previous study.[23] This 16-mer gapmer design was used in the present study to investigate whether varying sequence lengths led to changes in the effects of the modifications on T_m, potency, and acute CNS toxicity of the ASO. A series of ASOs, complementary RNAs designed to target mouse *Malat1* RNA and *Mapt* mRNA, was synthesized by Gene Design (Osaka, Japan). All PO linkages in the ASO sequences were replaced by PS linkages.

T_m analysis

T_m analysis of ASOs was done by measuring the UV absorbance using a Shimadzu TMSPC-8 Temperature Controlled Accessory (Shimadzu, Kyoto, Japan). Each sample containing 5 μM ASO and complementary RNAs in PBS (Nacalai Tesque, Kyoto, Japan) were annealed by heating at 95°C for 5 min and cooling slowly to room temperature. Then, ASO/complementary RNA duplexes were diluted to a final concentration of 2 μM with PBS. ASO melting curves were obtained at 260 nm by heating from 20°C to 95°C at 0.5°C min^{-1}. T_m values were calculated as the first derivative of each curve.

Cell culture and transfection of ASOs

The Neuro-2a mouse neuroblastoma cell line was cultured in DMEM (Wako, Osaka, Japan) supplemented with 10% fetal bovine serum (FBS) and 1% penicillin/streptomycin in an incubator at 37°C with 5% CO_2. A total of 3×10^4 cells were seeded in 48-well plates and transfected with increasing concentrations of ASOs using 5 μg/mL Lipofectamine 2000 (Invitrogen, Carlsbad, CA, USA) in a Gibco Opti-MEM medium (Thermo Fisher Scientific, Waltham, MA, USA). DMEM with FBS and penicillin/streptomycin were added to transfection mixes after 3 h. After incubation of cells for 48 h at 37°C with 5% CO_2, the cells were used in subsequent protocols.

RNA isolation and quantitative real-time PCR analysis

RNA was extracted from collected samples using ISOGEN I (Nippon Gene, Tokyo, Japan), following the manufacturer's procedure. cDNA was synthesized from the isolated total RNA using Takara 5× Prime Script RT Master Mix (Takara Bio, Inc., Kusatsu, Shiga, Japan). For the detection of RNA or mRNA expression, quantitative real-time PCR analyses were performed for mouse *Malat1*, *Mapt*, and actin beta (*Actb*; known as Β-actin) genes. This was done using a LightCycler 480 system with a LightCycler 480 probes master kit (Roche Diagnostics, Basel, Switzerland). The relative RNA or mRNA levels of the target genes were normalized to that of *Actb*. Primers and probes for mouse *Malat1* and *Mapt* were designed by TaqMan (Applied Biosystems, Foster City, CA, USA), while those for *Actb* were designed by Sigma-Aldrich (St. Louis, MO, USA). The real-time PCR was conducted following the Minimum Information for publication of real-time PCR Experiments guidelines.[49]

Mouse and ICV injection

In all experiments, 7-week-old wild-type female Crlj; CD1 mice purchased from Oriental Yeast (Tokyo, Japan) were used. The mice were anesthetized and maintained with 2% isoflurane throughout the procedure. The head was immobilized in a stereotaxic frame with a head holding adaptor and an auxiliary ear bar (Narishige, Tokyo, Japan). The skin of the skull was removed and the skull was swabbed with ethanol. A small burr hole was drilled in the skull using a 0.3-mm microdrill, positioned 1 mm to the left lateral from the midline and 0.2 mm posterior to the bregma. A 33G needle attached to a 25 μL glass syringe (Hamilton Company, Bonaduz, Switzerland) was inserted into the left ventricle of the brain through the burr hole. ASOs dissolved in PBS or PBS (used as a vehicle) were administered to mice (n = 4 per group) via ICV injection at a dose of 15 IL/3 min. Reagents against the sigma receptors were prepared with PBS buffer using 150.1 μg (0.43 μM) of BD 1063 dihydrochloride (Sigma-Aldrich) as the sigma-1 receptor antagonist, and 113.2 μg (0.25 μM) of SM21 maleate (ABCAM Limited, Castlebar, Mayo, Ireland) as the sigma-2 receptor antagonist.[30] For ASO injection with pretreatment of the antagonists, mice (n = 2–3 per group) were first administrated with the modulators at 10 mL/2 min. At 5 min after that, ASOs dissolved in PBS were administrated at 5 mL/1 min through the same burr hole. The needle was slowly withdrawn 5 min after administration, and the skin was closed with nylon sutures. The mice were subcutaneously injected with gentamycin (0.02 μg/kg) and carprofen (5 mg/kg). They recovered from anesthesia in the cage on a heating plate. Prior to euthanasia for post-mortem analyses, the mice were anesthetized with intraperitoneally injected pentobarbital (60 mg/kg) and transcardially perfused with PBS. All procedures were conducted in accordance with the ethics and safety guidelines for animal experimentation and were approved

by the Institutional Animal Care and Use Committee of Tokyo Medical and Dental University (No. A2022-085A).

Open-field test

The locomotor activity of mice was assessed using the open-field test. The open field consisted of a vinyl chloride gray square board and wall (50 cm × 50 cm × 40 cm; Muromachi Kikai, Tokyo, Japan). Mice were placed in the center of the floor and allowed to freely explore for 5 min. The movements were recorded using an automatic video tracking system (ANY-maze; Stoelting, IL, USA), and total distance, max speed, and mobile time were analyzed at 1 h after ICV injection. Prior to each test, the open field floor was cleaned with ethanol.

Acute toxicity scoring system

To evaluate abnormal neurobehaviors in mice, the acute toxicity scoring system was previously established.[22] The battery items in the acute toxicity scoring system were classified into five neurobehavioral categories: (1) consciousness, (2) motor function, (3) appearance, (4) hyperactivity, and (5) involuntary movement. Each category was scored on a 5-point scale, and the final acute tolerability score was calculated by summing up the scores from each category (minimum = 0, maximum = 20). Additionally, 22 points were given to mice that died during or after ICV administration. The animals were blindly scored by a scorer who did not administer ASOs at 0.5, 1, 2, 3, and 4 h after ICV administration.

Electrophoretic mobility shift assay

Electrophoresis was conducted using a 5%–20% SuperSep Ace gel (FUJIFILM Wako Pure Chemical Corporation, Osaka, Japan) with 1× TBE Running Buffer (from a 10× stock; Invitrogen, Carlsbad, CA, USA). Each sample, containing 3 μM Cy5-labeled ASO and human commercial pooled CSF (Analytical Biological Services, Wilmington, DE, USA), was incubated for 1 h at 37°C. Afterward, 6× loading buffer (Takara Bio, Inc., Shiga, Japan) was added. These samples, along with WIDE-VIEW Prestained Protein Size Marker III (FUJIFILM Wako Pure Chemical Corporation, Osaka, Japan) as the molecular marker, were loaded onto the gel. Electrophoresis was run for 90 min at 300 V and 20 mA. The gel was visualized using a ChemiDoc Touch Imaging System (BioRad, Hercules, CA, USA).

Statistical analysis

Pairwise and multiple comparisons were performed using Student's t-tests and one-way ANOVA with Tukey's multiple-comparison tests, respectively. Mean values and standard errors are presented in the figures. The statistical significance level was set at a p value of less than 0.05. Statistical analyses and IC_{50} value calculation by fitting the data to a sigmoidal dose-response curve using a defined top of 100% and bottom of 0% were conducted using GraphPad Prism 9 software (GraphPad Software, San Diego, CA, USA).

DATA AND CODE AVAILABILITY

The data supporting the findings of this study are available from the corresponding author upon reasonable request.

SUPPLEMENTAL INFORMATION

Supplemental information can be found online at https://doi.org/10.1016/j.omtn.2024.102161.

ACKNOWLEDGMENTS

This study was supported under grants JP21wm0525032 (to K.Y.), JP18am0301003, JP20am0401006, and JP21ae0121026 (to T.Yokota.) from AMED (Tokyo, Japan), JST FOREST Program (Japan; JPMJFR216H to K.Y.), JSPS KAKENHI Grant-in-Aid for Challenging Research (Exploratory) (JP20K21882), Scientific Research (B) (JP22H02979) from MEXT (Tokyo, Japan; to K.Y.), and a grant from the Takeda Science Foundation (to K.Y.). A part of this research was supported by AMED under Grant Number JP19am0401003 (to S.O.). The authors would like to thank Enago (http://www.enago.jp) for the English language review.

AUTHOR CONTRIBUTIONS

T.M., K.Y., S.S.L.M., M.K., C.J., T.Yamaguchi, R.I.H., and O.N. performed the experiments and analyzed data. K.Y., S.O., and T.Yokota conceived and supervised the study. T.M. wrote the manuscript. K.Y., T.N., S.O., and T.Yokota reviewed the manuscript.

DECLARATION OF INTERESTS

T.Yokota collaborates with Takeda Pharmaceuticals Co., Ltd., Daiichi Sankyo Co., Ltd., Rena Therapeutics Co., Ltd., Toray Industries Co., Ltd., Eisai Co., Ltd., and Sumitomo Pharma Co., Ltd., and serves as the academic adviser for Rena Therapeutics, Inc., and Braizon Therapeutics, Inc. A provisional patent application (PCT/JP2023/024244) describing the work and chemistry used in this study has been submitted (authors: T.Yokota, K.Y., T.M., S.O., and O.N.).

REFERENCES

1. Evers, M.M., Toonen, L.J.A., and van Roon-Mom, W.M.C. (2015). Antisense oligonucleotides in therapy for neurodegenerative disorders. Adv. Drug Deliv. Rev. *87*, 90–103.
2. Yamamoto, T., Nakatani, M., Narukawa, K., and Obika, S. (2011). Antisense drug discovery and development. Future Med. Chem. *3*, 339–365.
3. Kole, R., Krainer, A.R., and Altman, S. (2012). RNA therapeutics: beyond RNA interference and antisense oligonucleotides. Nat. Rev. Drug Discov. *11*, 125–140.
4. Teplova, M., Minasov, G., Tereshko, V., Inamati, G.B., Cook, P.D., Manoharan, M., and Egli, M. (1999). Crystal structure and improved antisense properties of 2'-O-(2-methoxyethyl)-RNA. Nat. Struct. Biol. *6*, 535–539.
5. Obika, S., Nanbu, D., Hari, Y., Andoh, J.-i., Morio, K.-i., Doi, T., and Imanishi, T. (1998). Stability and structural features of the duplexes containing nucleoside analogues with a fixed N-type conformation, 2′-O,4′-C-methyleneribonucleosides. Tetrahedron Lett. *39*, 5401–5404.
6. Vester, B., and Wengel, J. (2004). LNA (locked nucleic acid): high-affinity targeting of complementary RNA and DNA. Biochemistry *43*, 13233–13241.
7. Eckstein, F. (2014). Phosphorothioates, essential components of therapeutic oligonucleotides. Nucleic Acid Therapeut. *24*, 374–387.
8. Shadid, M., Badawi, M., and Abulrob, A. (2021). Antisense oligonucleotides: absorption, distribution, metabolism, and excretion. Expet Opin. Drug Metabol. Toxicol. *17*, 1281–1292.
9. Finkel, R.S., Mercuri, E., Darras, B.T., Connolly, A.M., Kuntz, N.L., Kirschner, J., Chiriboga, C.A., Saito, K., Servais, L., Tizzano, E., et al. (2017). Nusinersen versus Sham Control in Infantile-Onset Spinal Muscular Atrophy. N. Engl. J. Med. *377*, 1723–1732.

10. Mercuri, E., Darras, B.T., Chiriboga, C.A., Day, J.W., Campbell, C., Connolly, A.M., Iannaccone, S.T., Kirschner, J., Kuntz, N.L., Saito, K., et al. (2018). Nusinersen versus Sham Control in Later-Onset Spinal Muscular Atrophy. N. Engl. J. Med. *378*, 625–635.

11. Miller, T.M., Cudkowicz, M.E., Genge, A., Shaw, P.J., Sobue, G., Bucelli, R.C., Chiò, A., Van Damme, P., Ludolph, A.C., Glass, J.D., et al. (2022). Trial of antisense oligonucleotide tofersen for SOD1 ALS. N. Engl. J. Med. *387*, 1099–1110.

12. Tabrizi, S.J., Leavitt, B.R., Landwehrmeyer, G.B., Wild, E.J., Saft, C., Barker, R.A., Blair, N.F., Craufurd, D., Priller, J., Rickards, H., et al. (2019). Targeting huntingtin expression in patients with Huntington's disease. N. Engl. J. Med. *380*, 2307–2316.

13. Mummery, C.J., Börjesson-Hanson, A., Blackburn, D.J., Vijverberg, E.G.B., De Deyn, P.P., Ducharme, S., Jonsson, M., Schneider, A., Rinne, J.O., Ludolph, A.C., et al. (2023). Tau-targeting antisense oligonucleotide MAPTRx in mild Alzheimer's disease: a phase 1b, randomized, placebo-controlled trial. Nat. Med. *29*, 1437–1447.

14. Goyenvalle, A., Jimenez-Mallebrera, C., van Roon, W., Sewing, S., Krieg, A.M., Arechavala-Gomeza, V., and Andersson, P. (2023). Considerations in the Preclinical Assessment of the Safety of Antisense Oligonucleotides. Nucleic Acid Therapeut. *33*, 1–16.

15. Miller, T., Cudkowicz, M., Shaw, P.J., Andersen, P.M., Atassi, N., Bucelli, R.C., Genge, A., Glass, J., Ladha, S., Ludolph, A.L., et al. (2020). Phase 1-2 trial of antisense oligonucleotide tofersen for SOD1 ALS. N. Engl. J. Med. *383*, 109–119.

16. Kishimoto, Y., Nakagawa, O., Fujii, A., Yoshioka, K., Nagata, T., Yokota, T., Hari, Y., and Obika, S. (2021). 2',4'-BNA/LNA with 9-(2-aminoethoxy)-1,3-diaza-2-oxophenoxazine efficiently forms duplexes and has enhanced enzymatic resistance. Chemistry *27*, 2427–2438.

17. Flanagan, W.M., Wolf, J.J., Olson, P., Grant, D., Lin, K.Y., Wagner, R.W., and Matteucci, M.D. (1999). A cytosine analog that confers enhanced potency to antisense oligonucleotides. Proc. Natl. Acad. Sci. USA *96*, 3513–3518.

18. Stanton, R., Sciabola, S., Salatto, C., Weng, Y., Moshinsky, D., Little, J., Walters, E., Kreeger, J., DiMattia, D., Chen, T., et al. (2012). Chemical modification study of antisense gapmers. Nucleic Acid Therapeut. *22*, 344–359.

19. Post, N., Yu, R., Greenlee, S., Gaus, H., Hurh, E., Matson, J., and Wang, Y. (2019). Metabolism and Disposition of Volanesorsen, a 2′-O-(2 methoxyethyl) Antisense Oligonucleotide, Across Species. Drug Metab. Dispos. *47*, 1164–1173.

20. Gaus, H.J., Gupta, R., Chappell, A.E., Østergaard, M.E., Swayze, E.E., and Seth, P.P. (2019). Characterization of the interactions of chemically-modified therapeutic nucleic acids with plasma proteins using a fluorescence polarization assay. Nucleic Acids Res. *47*, 1110–1122.

21. Liang, X.H., Shen, W., Sun, H., Kinberger, G.A., Prakash, T.P., Nichols, J.G., and Crooke, S.T. (2016). Hsp90 protein interacts with phosphorothioate oligonucleotides containing hydrophobic 2'-modifications and enhances antisense activity. Nucleic Acids Res. *44*, 3892–3907.

22. Jia, C., Lei Mon, S.S., Yang, Y., Katsuyama, M., Yoshida-Tanaka, K., Nagata, T., Yoshioka, K., and Yokota, T. (2023). Change of intracellular calcium level causes acute neurotoxicity by antisense oligonucleotides via CSF route. Mol. Ther. Nucleic Acids *31*, 182–196.

23. Hagedorn, P.H., Brown, J.M., Easton, A., Pierdomenico, M., Jones, K., Olson, R.E., Mercer, S.E., Li, D., Loy, J., Høg, A.M., et al. (2022). Acute neurotoxicity of antisense oligonucleotides after intracerebroventricular injection into mouse brain can be predicted from sequence features. Nucleic Acid Therapeut. *32*, 151–162.

24. Hayashi, T., and Su, T.P. (2007). Sigma-1 receptor chaperones at the ER-mitochondrion interface regulate Ca(2+) signaling and cell survival. Cell *131*, 596–610.

25. Lachance, V., Bélanger, S.M., Hay, C., Le Corvec, V., Banouvong, V., Lapalme, M., Tarmoun, K., Beaucaire, G., Lussier, M.P., and Kourrich, S. (2023). Overview of Sigma-1R subcellular specific biological functions and role in neuroprotection. Int. J. Mol. Sci. *24*, 1971.

26. Rousseaux, C.G., and Greene, S.F. (2016). Sigma receptors [σRs]: biology in normal and diseased states. J. Recept. Signal Transduct. Res. *36*, 327–388.

27. Nakagawa, O., Ming, X., Huang, L., and Juliano, R.L. (2010). Targeted intracellular delivery of antisense oligonucleotides via conjugation with small-molecule ligands. J. Am. Chem. Soc. *132*, 8848–8849.

28. Donnier-Maréchal, M., Larchanché, P.E., Le Broc, D., Furman, C., Carato, P., and Melnyk, P. (2015). Carboline- and phenothiazine-derivated heterocycles as potent SIGMA-1 protein ligands. Eur. J. Med. Chem. *89*, 198–206.

29. Yamaji, R., Nakagawa, O., Kishimoto, Y., Fujii, A., Matsumura, T., Nakayama, T., Kamada, H., Osawa, T., Yamaguchi, T., and Obika, S. (2022). Synthesis and physical and biological properties of 1,3-diaza-2-oxophenoxazine-conjugated oligonucleotides. Bioorg. Med. Chem. *72*, 116972.

30. Kotagale, N.R., Mendhi, S.M., Aglawe, M.M., Umekar, M.J., and Taksande, B.G. (2013). Evidences for the involvement of sigma receptors in antidepressant like effect of quetiapine in mice. Eur. J. Pharmacol. *702*, 180–186.

31. Bondensgaard, K., Petersen, M., Singh, S.K., Rajwanshi, V.K., Kumar, R., Wengel, J., and Jacobsen, J.P. (2000). Structural studies of LNA:RNA duplexes by NMR: conformations and implications for RNase H activity. Chemistry *6*, 2687–2695.

32. Ortega, J.A., Blas, J.R., Orozco, M., Grandas, A., Pedroso, E., and Robles, J. (2007). Binding affinities of oligonucleotides and PNAs containing phenoxazine and G-clamp cytosine analogues are unusually sequence-dependent. Org. Lett. *9*, 4503–4506.

33. Aartsma-Rus, A., van Vliet, L., Hirschi, M., Janson, A.A.M., Heemskerk, H., de Winter, C.L., de Kimpe, S., van Deutekom, J.C.T., 't Hoen, P.A., and van Ommen, G.J.B. (2009). Guidelines for antisense oligonucleotide design and insight into splice-modulating mechanisms. Mol. Ther. *17*, 548–553.

34. Chan, J.H.P., Lim, S., and Wong, W.S.F. (2006). Antisense oligonucleotides: from design to therapeutic application. Clin. Exp. Pharmacol. Physiol. *33*, 533–540.

35. Papargyri, N., Pontoppidan, M., Andersen, M.R., Koch, T., and Hagedorn, P.H. (2020). Chemical diversity of locked nucleic acid-modified antisense oligonucleotides allows optimization of pharmaceutical properties. Mol. Ther. Nucleic Acids *19*, 706–717.

36. Yamamoto, T., Yasuhara, H., Wada, F., Harada-Shiba, M., Imanishi, T., and Obika, S. (2012). Superior Silencing by 2',4'-BNA(NC)-Based Short Antisense Oligonucleotides Compared to 2',4'-BNA/LNA-Based Apolipoprotein B Antisense Inhibitors. J. Nucleic Acids *2012*, 707323.

37. Yamamoto, T., Fujii, N., Yasuhara, H., Wada, S., Wada, F., Shigesada, N., Harada-Shiba, M., and Obika, S. (2014). Evaluation of multiple-turnover capability of locked nucleic acid antisense oligonucleotides in cell-free RNase H-mediated antisense reaction and in mice. Nucleic Acid Therapeut. *24*, 283–290.

38. Bailey, J.K., Shen, W., Liang, X.H., and Crooke, S.T. (2017). Nucleic acid binding proteins affect the subcellular distribution of phosphorothioate antisense oligonucleotides. Nucleic Acids Res. *45*, 10649–10671.

39. Crooke, S.T., Vickers, T.A., and Liang, X.H. (2020). Phosphorothioate modified oligonucleotide-protein interactions. Nucleic Acids Res. *48*, 5235–5253.

40. Shen, W., De Hoyos, C.L., Migawa, M.T., Vickers, T.A., Sun, H., Low, A., Bell, T.A., Rahdar, M., Mukhopadhyay, S., Hart, C.E., et al. (2019). Chemical modification of PS-ASO therapeutics reduces cellular protein-binding and improves the therapeutic index. Nat. Biotechnol. *37*, 640–650.

41. Brailoiu, E., Chakraborty, S., Brailoiu, G.C., Zhao, P., Barr, J.L., Ilies, M.A., Unterwald, E.M., Abood, M.E., and Taylor, C.W. (2019). Choline Is an Intracellular Messenger Linking Extracellular Stimuli to IP3-Evoked Ca2+ Signals through Sigma-1 Receptors. Cell Rep. *26*, 330–337.e4.

42. Kume, T., Nishikawa, H., Taguchi, R., Hashino, A., Katsuki, H., Kaneko, S., Minami, M., Satoh, M., and Akaike, A. (2002). Antagonism of NMDA receptors by sigma receptor ligands attenuates chemical ischemia-induced neuronal death in vitro. Eur. J. Pharmacol. *455*, 91–100.

43. Qiu, J., Wu, L., Qu, R., Jiang, T., Bai, J., Sheng, L., Feng, P., and Sun, J. (2022). History of development of the life-saving drug "Nusinersen" in spinal muscular atrophy. Front. Cell. Neurosci. *16*, 942976.

44. Scoles, D.R., Meera, P., Schneider, M.D., Paul, S., Dansithong, W., Figueroa, K.P., Hung, G., Rigo, F., Bennett, C.F., Otis, T.S., and Pulst, S.M. (2017). Antisense oligonucleotide therapy for spinocerebellar ataxia type 2. Nature *544*, 362–366.

45. Toonen, L.J.A., Casaca-Carreira, J., Pellisé-Tintoré, M., Mei, H., Temel, Y., Jahanshahi, A., and van Roon-Mom, W.M.C. (2018). Intracerebroventricular Administration of a 2'-O-Methyl Phosphorothioate Antisense Oligonucleotide Results in Activation of the Innate Immune System in Mouse Brain. Nucleic Acid Therapeut. *28*, 63–73.

46. Pollak, A.J., Zhao, L., and Crooke, S.T. (2023). Systematic Analysis of Chemical Modifications of Phosphorothioate Antisense Oligonucleotides that Modulate Their Innate Immune Response. Nucleic Acid Therapeut. *33*, 95–107.

47. Hung, G., Xiao, X., Peralta, R., Bhattacharjee, G., Murray, S., Norris, D., Guo, S., and Monia, B.P. (2013). Characterization of target mRNA reduction through in situ RNA hybridization in multiple organ systems following systemic antisense treatment in animals. Nucleic Acid Therapeut. *23*, 369–378.

48. Saini, A., and Chawla, P.A. (2023). Breaking barriers with tofersen: Enhancing therapeutic opportunities in amyotrophic lateral sclerosis. Eur. J. Neurol. *31*, e16140.

49. Bustin, S.A., Benes, V., Garson, J.A., Hellemans, J., Huggett, J., Kubista, M., Mueller, R., Nolan, T., Pfaffl, M.W., Shipley, G.L., et al. (2009). The MIQE guidelines: minimum information for publication of quantitative real-time PCR experiments. Clin. Chem. *55*, 611–622.

OMTN, Volume *35*

Supplemental information

Favorable efficacy and reduced acute neurotoxicity by antisense oligonucleotides with 2′,4′-BNA/LNA with 9-(aminoethoxy)phenoxazine

Taiki Matsubayashi, Kotaro Yoshioka, Su Su Lei Mon, Maho Katsuyama, Chunyan Jia, Takao Yamaguchi, Rintaro Iwata Hara, Tetsuya Nagata, Osamu Nakagawa, Satoshi Obika, and Takanori Yokota

Table S1. Abbreviation in this study.

Abbreviation	Spell out
**Gene name**	
Actb	actin beta
Malat1	metastasis associated in lung adenocarcinoma transcript-1
Mapt	microtubule-associated protein tau
**Chemical modification**	
2',4'-BNA/LNA	2'-O,4'-C-methylene-bridged nucleic acid/locked nucleic acid
BNAP-AEO	2',4'-BNA/LNA with a 9-(aminoethoxy)phenoxazine
G-clamp	9-(aminoethoxy)phenoxazine
MOE	2'O-methoxyethyl RNA
PO linkage	phosphodiester linkage
PS linkage	phosphorothioate linkage
**Others**	
ASO	antisense oligonucleotide
CNS	central nervous system
CSF	cerebrospinal fluid
DMEM	Dulbecco's modified minimal essential medium
FBS	fetal bovine serum
FDA	US Food and Drug Administration
ICV	intracerebroventricular
T_m	melting temperature

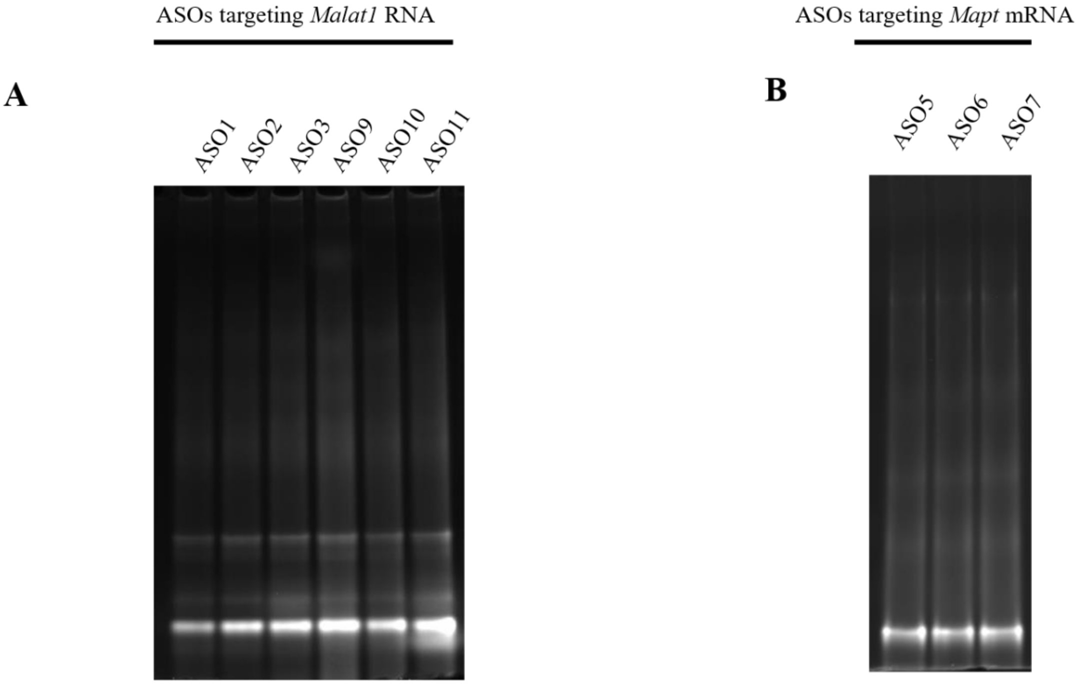

Figure S1. Electrophoretic mobility shift assay of ASOs targeting mouse *Malat1* RNA and *Mapt* mRNA.

(A) Interaction of 3 µM Cy5-labeled ASOs targeting *Malat1* RNA (ASO1, ASO2, ASO3, ASO9, ASO10, and ASO11) with proteins in human CSF. (B) Interaction of 3 µM Cy5-labeled ASOs targeting *Mapt* mRNA (ASO5, ASO6, and ASO7) with proteins in human CSF.

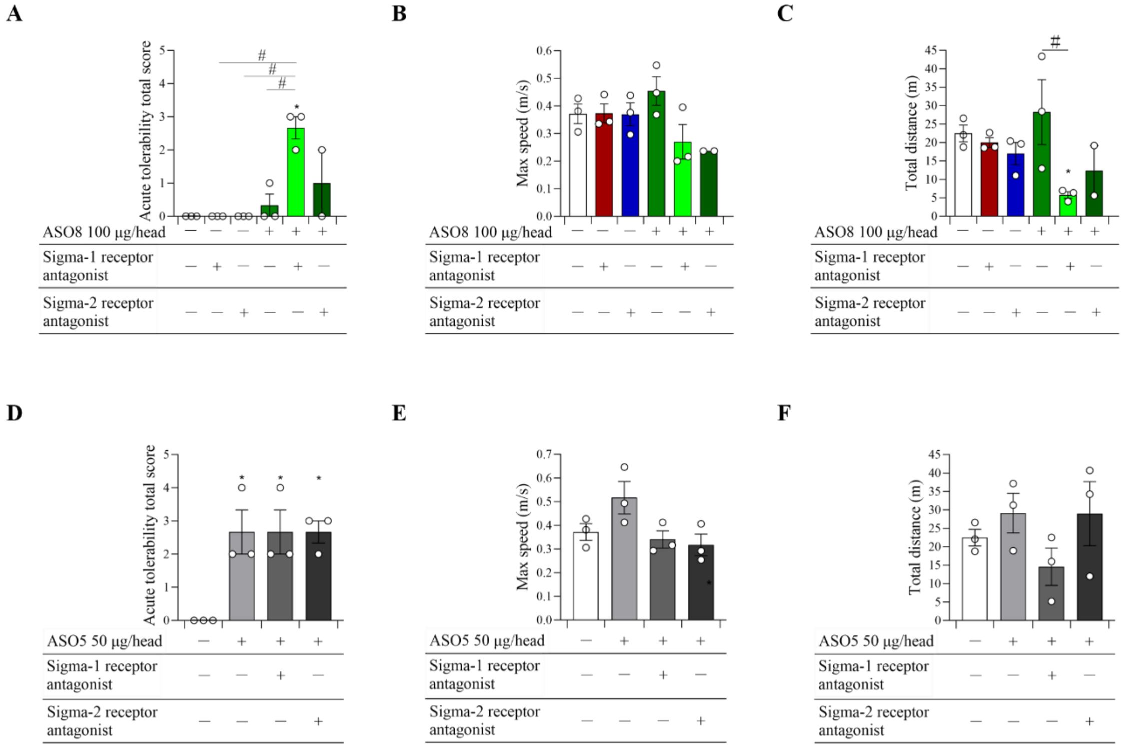

Figure S2. Assessment of acute CNS toxicity by ICV injection of 16-mer ASOs after pretreatment with the sigma-1 and -2 receptor antagonist.

(A) Acute tolerability scores in mice 1 h after the ICV injection of PBS as a negative control, 0.43 µmol sigma-1 receptor antagonist (BD 1063 dihydrochloride), 0.25 µmol sigma-2 receptor antagonist (SM21 maleate), and 19.0 nmol 16-mer ASO with 2 BNAP-AEO (ASO8; 100 µg/head) with pretreatment of PBS, sigma-1 receptor antagonist, or sigma-2 receptor antagonist. (B, C) Locomotor activity parameters, including maximum speed (B) and total distance (C), of mice shown in (A) 1 h after the ICV injection. (D) Acute tolerability scores in mice 1 h after the ICV injection of PBS and 9.5 nmol 16-mer ASO including only DNA (ASO5; 50 µg/head) with pretreatment of PBS, 0.43 µmol sigma-1 receptor antagonist, or 0.25 µmol sigma-2 receptor antagonist. (E, F) Locomotor activity parameters, including maximum speed (E) and total distance (F), of mice shown in (D) 1 h after the ICV injection.

Data are presented as mean ± standard error (n = 2-3 per group). *$p < 0.05$; data were analyzed using paired t-test with PBS. # < 0.05; data were analyzed using one-way ANOVA, followed by Tukey's post hoc test.

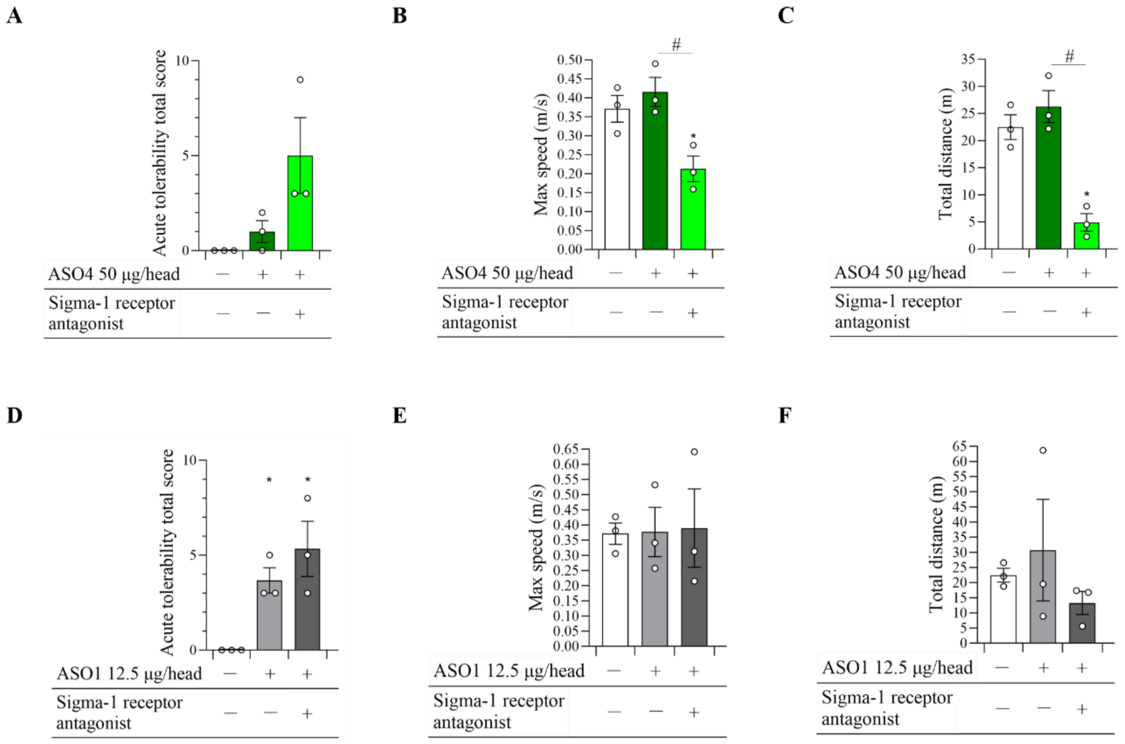

Figure S3. Assessment of acute CNS toxicity by ICV injection of the 20-mer ASO after pretreatment with the sigma-1 receptor antagonist.

(A) Acute tolerability scores in mice 1 h after the ICV injection of PBS as a negative control and 7.63 nmol 20-mer ASO with 2 BNAP-AEO (ASO4; 50 μg/head) with pretreatment of PBS or 0.43 μmol sigma-1 receptor antagonist (BD 1063 dihydrochloride). (B, C) Locomotor activity parameters, including maximum speed (B) and total distance (C), of mice shown in (A) 1 h after the ICV injection. (D) Acute tolerability scores in mice 1 h after the ICV injection of PBS and 1.91 nmol 20-mer ASO including only DNA (ASO1; 12.5 μg/head) with pretreatment of PBS or 0.43 μmol sigma-1 receptor antagonist. (E, F) Locomotor activity parameters, including maximum speed (E) and total distance (F), of mice shown in (D) 1 h after the ICV injection.

Data are presented as mean ± standard error (n = 3 per group). *$p < 0.05$; data were analyzed using paired t-test with PBS. # < 0.05; data were analyzed using one-way ANOVA, followed by Tukey's post hoc test.

Video S1. Mitigation of acute CNS toxicity by ICV injection of G-clamp-modified ASOs targeting *Malat1* RNA.

(A) Video of mouse was recorded 1 hour after ICV injection of PBS. (B) Video of mouse was recorded 1 hour after ICV injection of 7.63 nmol 20-mer ASO including only DNA (ASO1). (C) Video of mouse was recorded 1 hour after ICV injection of 7.63 nmol 20-mer ASO with 2 LNA (ASO2). (D) Video of mouse was recorded 1 hour after ICV injection of 7.63 nmol 20-mer ASO with 2 G-clamp (ASO3). (E) Video of mouse was recorded 1 hour after ICV injection of 7.63 nmol 20-mer ASO with 2 BNAP-AEO (ASO4).

Video S2. Mitigation of acute CNS toxicity by ICV injection of G-clamp-modified ASOs with MOE targeting *Malat1* RNA.

(A) Video of mouse was recorded 1 hour after ICV injection of 7.63 nmol 20-mer gapmer ASO with 5 and 4 MOE at the 5' and 3' ends (ASO9). (B) Video of mouse was recorded 1 hour after ICV injection of 7.63 nmol 20-mer gapmer ASO with 4 MOE and 1 LNA at the 5' end and 3 MOE and 1 LNA at the 3' end (ASO10). (C) Video of mouse was recorded 1 hour after ICV injection of 7.63 nmol 20-mer gapmer ASO with 4 MOE and 1 G-clamp at the 5' end and 3 MOE and 1 G-clamp at the 3' end (ASO11). (D) Video of mouse was recorded 1 hour after ICV injection of 7.63 nmol 20-mer gapmer ASO with 4 MOE and 1 BNAP-AEO at the 5' end and 3 MOE and 1 BNAP-AEO at the 3' end (ASO12).

Video S3. Mitigation of acute CNS toxicity by ICV injection of G-clamp-modified ASOs targeting *Mapt* mRNA.

(A) Video of mouse was recorded 1 hour after ICV injection of 19.0 nmol 16-mer ASO including only DNA (ASO5). (B) Video of mouse was recorded 1 hour after ICV injection of 19.0 nmol 16-mer ASO with 2 LNA (ASO6). (C) Video of mouse was recorded 1 hour after ICV injection of 19.0 nmol 16-mer ASO with 2 G-clamp (ASO7). (D) Video of mouse was recorded 1 hour after ICV injection of 19.0 nmol 16-mer ASO with 2 BNAP-AEO (ASO8).

Video S4. Acute CNS toxicity of mice by ICV injection of the 16-mer ASOs with presence or absence of pretreatments of the sigma receptor antagonists.

Videos were recorded at 1 hour after ICV injection of 0.43 μmol sigma-1 receptor antagonist (A), or 0.25 μmol sigma-2 receptor antagonist (B). Videos were recorded at 1 hour after ICV injection of 19.0 nmol 16-mer ASO with 2 BNAP-AEO (ASO8) with pretreatment of PBS (C), the sigma-1 receptor antagonist (D), or the sigma-2 receptor antagonist (E). Videos were recorded 1 hour after ICV injection of 9.5 nmol 16-mer ASO including only DNA (ASO5) with pretreatment of PBS (F), the sigma-1 receptor antagonist (G), or the sigma-2 receptor antagonist (H).

Video S5. Acute CNS toxicity of mice by ICV injection of the 20-mer ASOs with presence or absence of pretreatments of the sigma receptor antagonists.

Videos were recorded 1 hour after ICV injection of 7.63 nmol 20-mer ASO with 2 BNAP-AEO (ASO4) with pretreatment of PBS (A) or the sigma-1 receptor antagonist (B). Videos were recorded 1 hour after ICV injection of 1.91 nmol 20-mer ASO including only DNA (ASO1) with pretreatment of PBS (C) or the sigma-1 receptor antagonist (D).
